# Supplementary material for: Intrathecal or intravenous AAV9-IDUA/RGX-111 at minimal effective dose prevents cardiac, skeletal and neurologic manifestations of murine MPS I
Source: Mol Ther Methods Clin Dev. 2024 Nov 4;32(4):101369. doi: 10.1016/j.omtm.2024.101369 (PMC11646787; doi:10.1016/j.omtm.2024.101369)
Supplement: Document S1. Figures S1–S4 [file mmc1.pdf]

OMTM, Volume *32*

Supplemental information

Intrathecal or intravenous AAV9-IDUA/RGX-111 at minimal effective dose prevents cardiac, skeletal and neurologic manifestations of murine MPS I

Lalitha R. Belur, Avery K. Huber, Hillary Mantone, Mason Robertson, Miles C. Smith, Andrea D. Karlen, Kelley F. Kitto, Li Ou, Chester B. Whitley, Elizabeth Braunlin, Justin Furcich, Troy C. Lund, Davis Seelig, Carolyn A. Fairbanks, Nicholas Buss, Kwi Hye Kim, and R. Scott McIvor

SUPPLEMENTAL FIGURES.

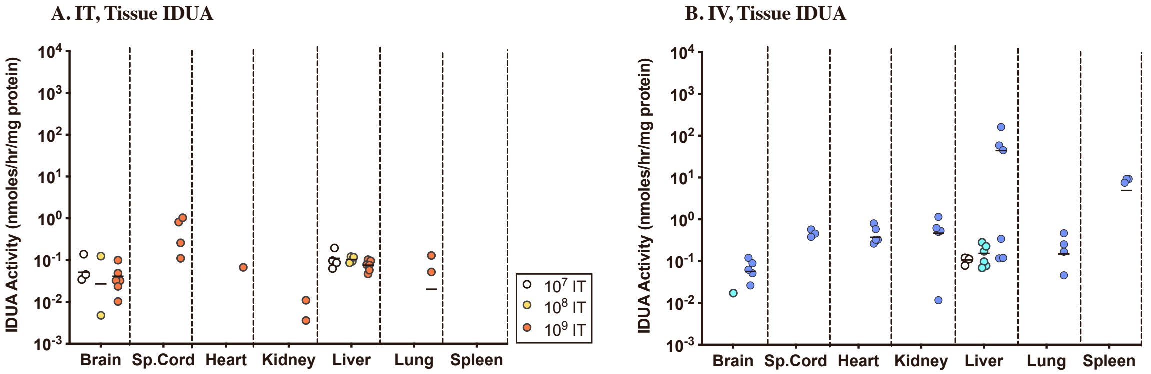

Figure S1. Tissue IDUA activities in MPS mice administered 10^7-10^9 vg RGX-111. ***(Most related to Figure 2; Tissue IDUA Activities)*** **(A)** Intrathecal (IT) administration **(B)** Intravenous (IV) administration. Each data point indicates a value from a single animal. Tissues are indicated across the bottom of the graph. Route of administration or control group are indicated in the key. N = 6 (3 males and 3 females).

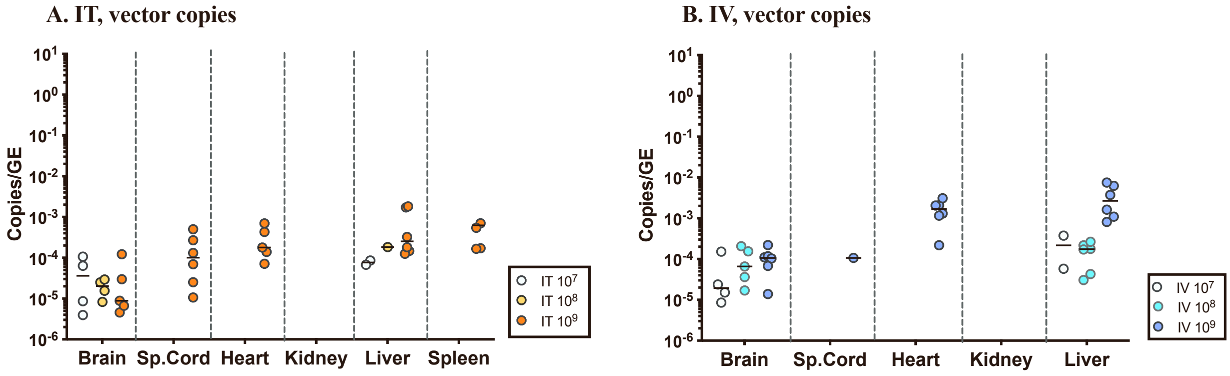

Figure S2. Biodistribution by qPCR for IDUA vector sequences ***(Most related to Figure 2; Vector distribution by qPCR).*** Tissue DNA extracts from animals treated with 10^7-10^9 vg RGX-111 either IT **(A)** or IV **(B)** were assayed for the presence of IDUA sequences by quantitative PCR. Dose is indicated in the key. Each symbol represents 1 animal, with the mean indicated by a horizontal bar. The lower limit of detection was determined by analysis of genomic DNA samples collected from heterozygote controls (<0.001 vector copies/genome equivalent).

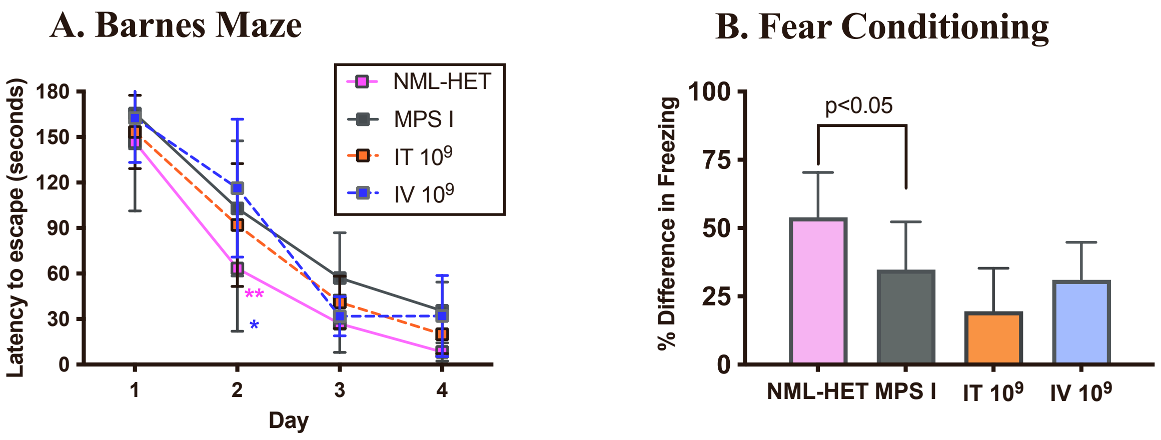

Figure S3. Effect of RGX-111 treatment on neurocognition *(Most related to Figure 3; Barnes Maze and Fear Conditioning)*. Animals treated with 10^9 vg RGX-111 either IT or IV were assayed for neurobehavior. **(A) Barnes maze.** The Barnes maze was used to assess the effect of vector administration on spatial learning and memory. Testing was carried out in four trials per day over 4 days for all groups (N=6) **P<.01 *P<.05. Mean latency to escape for the 4 trials is a shown for each day. **(B) Fear conditioning.** All groups were evaluated for fear conditioned response. Mean freezing time as a cued response is shown (N=6).

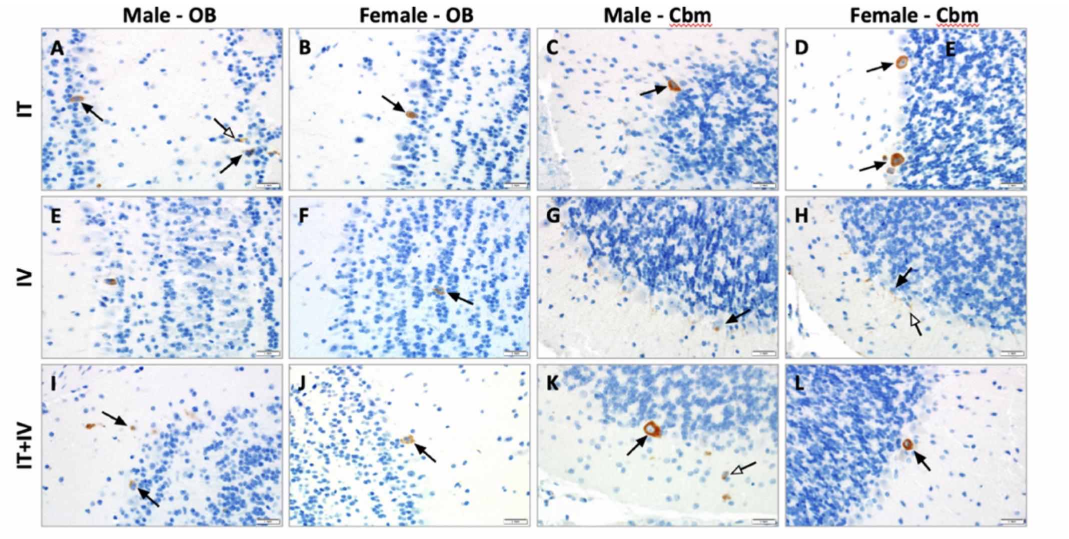

Figure S4. Similar density of IDUA-positive cells in the brains of male and female mice treated with 10^{10} vg RGX-111 (*Most related to Figure 7; IDUA-positive cells in livers of male and female mice*). Irrespective of sex, all treated mice demonstrate low numbers of IDUA-positive neurons (black arrows) and presumed glial cells (white arrows). Representative images from the olfactory bulb (OB) and the cerebellum (Cbm). Scale bars: A-L = 20 µm
