# Supplementary material for: Intrathecal or intravenous AAV9-IDUA/RGX-111 at minimal effective dose prevents cardiac, skeletal and neurologic manifestations of murine MPS I
Source: Mol Ther Methods Clin Dev. 2024 Nov 4;32(4):101369. doi: 10.1016/j.omtm.2024.101369 (PMC11646787; doi:10.1016/j.omtm.2024.101369)
Supplement: Document S2. Article plus supplemental information [file mmc2.pdf]

Molecular Therapy
Methods & Clinical Development
Original Article

Intrathecal or intravenous AAV9-IDUA/RGX-111 at minimal effective dose prevents cardiac, skeletal and neurologic manifestations of murine MPS I

Lalitha R. Belur,[1] Avery K. Huber,[1] Hillary Mantone,[1] Mason Robertson,[1] Miles C. Smith,[1] Andrea D. Karlen,[1] Kelley F. Kitto,[4] Li Ou,[2] Chester B. Whitley,[2] Elizabeth Braunlin,[2] Justin Furcich,[2] Troy C. Lund,[2] Davis Seelig,[3] Carolyn A. Fairbanks,[4] Nicholas Buss,[5] Kwi Hye Kim,[5] and R. Scott McIvor[1]

[1]Center for Genome Engineering, Department of Genetics, Cell Biology and Development, University of Minnesota, Minneapolis, MN, USA; [2]Department of Pediatrics, University of Minnesota, Minneapolis, MN, USA; [3]Comparative Pathology Shared Resource, University of Minnesota, Minneapolis, MN, USA; [4]Department of Pharmaceutics, University of Minnesota, Minneapolis MN, USA; [5]REGENXBIO, Inc., Rockville, MD, USA

Mucopolysaccharidosis type I (MPS I) is a rare metabolic disorder caused by deficiency of α-L-iduronidase (IDUA), resulting in glycosaminoglycan (GAG) accumulation and multisystemic disease. Current treatments include hematopoietic stem cell transplantation and enzyme replacement therapy, but these do not address all manifestations of the disease. We infused MPS I mice with an adeno-associated virus 9 (AAV9)-IDUA vector (RGX-111) at doses from 10^7 to 10^{10} vector genomes (vg) via intrathecal (IT), intravenous (IV), and intrathecal+intravenous (IT+IV) routes of administration. In mice administered doses $\leq 10^9$ vg IT or $\leq 10^8$ vg IV, there was no therapeutic benefit, while in mice administered 10^9 vg IV, there was a variable increase in IDUA activity with inconclusive neurocognitive and cardiac assessments. However, at the 10^{10} vg dose, we observed substantial metabolic correction, with restored IDUA levels and normalized tissue GAGs for all treatment groups. Aortic insufficiency was mostly normalized, neurologic deficit was prevented, and microcomputed tomography (micro-CT) analysis showed normalization of skeletal parameters. Histologic analysis showed minimal GAG storage and lysosomal pathology. We thus report a minimal effective dose of 10^{10} vg (5×10^{11} per kg) RGX-111 for IV and IT routes of administration in MPS I mice, which prevented neurocognitive deficit, cardiac insufficiency, and skeletal manifestations, as a model for genetic therapy of human MPS I.

INTRODUCTION

Mucopolysaccharidosis type 1 (MPS I) is an autosomal recessive lysosomal disorder caused by deficiency of the enzyme α-L-iduronidase (IDUA) resulting in an accumulation of the glycosaminoglycans (GAG) heparan sulfate and dermatan sulfate.[1–3] MPS I subjects suffer from cardiac defects, skeletal dysplasias, developmental delay, and hepatosplenomegaly.[4–6] The incidence of severe MPS I (Hurler syndrome) is approximately 1 in 100,000, while the incidence of the less severe form of MPS I (Scheie syndrome) is about 1 in 500,000 newborns. Current treatments for MPS I include enzyme replacement therapy (ERT) for attenuated disease and hematopoietic stem cell transplant (HSCT) for Hurler syndrome.[7–15] ERT has shown clinical improvement for peripheral manifestations of the disease but does not normalize GAG levels in all tissues such as bones and joints and is ineffective against neurological degeneration since the enzyme does not cross the blood-brain barrier.[7–12] As a result, ERT is unable to prevent the development of severe skeletal abnormalities, organ damage, or neurological complications, leading to persistent tissue damage and ongoing symptoms. The process is also expensive and requires time-consuming weekly or bimonthly infusions. HSCT relieves many manifestations of the disease and slows progression of neurocognitive decline due to donor cell engraftment.[10–15] However, the procedure is associated with significant mortality due to graft versus host disease (GVHD), and while donor-derived cells migrate to the brain and serve as a source of IDUA, patients continue to exhibit below-normal IQs.[16] There is thus a critical need for improved therapeutic approaches.

Recently, there have been substantial advances in the development of genetic therapies for lysosomal diseases, including *ex vivo* lentiviral transduced hematopoietic stem cells[17,18] and direct *in vivo* administration of adeno-associated viral (AAV) vectors (ClinicalTrials.gov: NCT03580083). The prospective benefit of genetic therapy is continuous expression of the missing gene product, thus providing a consistent source of enzyme for metabolic correction. Vectors or cells can also be administered in a way that directs virus transduction to specific target sites, leading to enzyme expression that is most beneficial for treatment of the disease. AAV vectors, including AAV9, have been extensively tested in preclinical studies for the treatment of MPS I.[19–32] AAV vectors have been effectively delivered via (1)

Received 24 June 2024; accepted 31 October 2024;
https://doi.org/10.1016/j.omtm.2024.101369.

Correspondence: Lalitha R. Belur, Center for Genome Engineering, Department of Genetics, Cell Biology and Development, University of Minnesota, Minneapolis, MN, USA.
E-mail: belur001@umn.edu

Published by Elsevier Inc. on behalf of The American Society of Gene and Cell Therapy.
This is an open access article under the CC BY license (http://creativecommons.org/licenses/by/4.0/).

Table 1. Groups, routes, and doses of RGX-111 administration

Genotype	Route of AAV treatment	Dose of RGX-111
MPS I	intrathecal (IT)	10^7, 10^8, 10^9, 10^{10} vg
MPS I	intravenous (IV)	10^7, 10^8, 10^9, 10^{10} vg
MPS I	intrathecal+intravenous (IT+IV)	10^{10} vg
NML-HET (Normal-Heterozygote)	no treatment	no vector (normal control)
MPS I	no treatment	no vector (affected control)

intraparenchymal (IP)[33–37]; (2) intra-cerebrospinal fluid (CSF), including intracerebroventricular (ICV), intracisterna magna (ICM), and intrathecal (IT)[19,20,28,29,32,38–41]; and (3) intravenous (IV)[22,42–45] routes of administration. Relative advantages of these different routes of vector administration include delivery of smaller volumes (IP), widespread delivery to the brain (ICV, ICM, IT), and non-invasiveness (IV). Relative disadvantages include invasiveness (IP, ICV), potential for tissue damage (IP, ICV; ICM has the potential for tissue damage if not administered correctly), and amount of vector needed to effectively transduce the brain (IV).

For MPS I, we and several groups have reported the effectiveness of AAV9 transducing the human *Idua* (h*Idua*) gene delivered via ICV, IT, intranasal, and IV routes of administration at high doses, variably demonstrating prevention of metabolic, histologic, skeletal, and neurologic disease.[19,20,28,29,32] However, the dose of AAV9-IDUA required to prevent the emergence of disease is less well characterized. The goal of the current study was to determine the relative dose effectiveness of AAV9-IDUA delivered systemically (IV) or through the CSF (IT) in preventing the emergence of both peripheral and neurologic manifestations of disease. Secondly, a substantial amount of AAV vector is released into the circulation after delivery through the CSF,[40,46,47] so a second goal of the current study was to determine the peripheral effectiveness of vector systemically redistributed after IT injection and the necessity of supplementing with additional IV-administered vector to achieve an efficacious outcome. As described below, we found that a surprisingly modest minimally effective dose of 10^{10} vector genomes (5×10^{11} per kg) administered IV or IT in 2-month-old MPS I mice was sufficient to prevent the subsequent emergence of metabolic, neurologic, cardiac, and skeletal manifestations of disease. These results contribute substantially to our understanding of AAV vector pharmacodynamics in the treatment of MPS I and mucopolysaccharidoses in general, as well as other diseases potentially treatable by AAV-mediated gene transfer.

RESULTS

Plasma IDUA activity and effect on urinary GAG excretion after IV, IT, or IT+IV RGX-111 administration

A schematic of the experimental groups is depicted in Table 1.

To reduce the likelihood of an immune response to the human transgene product, MPS I mice were immunosuppressed with cyclophosphamide (CP) and treated with RGX-111 ranging in dose from 10^7 to 10^{10} vg administered IT, IV, or IT+IV (10^{10} vg dose only). All experimental animals, including affected MPS I controls and normal healthy controls, were immunosuppressed with CP. In five of the female animals, CP administration did not prevent a rapid loss of IDUA enzyme activity, likely due to an immune response, and these animals were removed from the study. The vector schematic is depicted in Figure 1A. Blood was collected and processed into plasma starting at 2 weeks post-injection and at every subsequent month thereafter until the mice were humanely euthanized at 6–9 months post-AAV treatment. Plasma IDUA activities in males (Figure 1B) and females (Figure 1C) are graphed separately due to significant differences between the two. Plasma enzyme activity was undetectable in animals administered 10^7 vg RGX-111 IT or IV and in females administered 10^8 vg RGX-111 IV or IT. Low levels of plasma enzyme activity were detected in males injected with 10^8 vg RGX-111 that became extinguished 2 months after vector administration in IT- and IV-treated mice. The same observations were made in male and female mice treated with 10^9 vg IT, but in males treated with 10^9 vg IV, IDUA activity in plasma was 100 times the level of normal heterozygotes and was sustained for 4 months, at which time animals at this dose were euthanized. Female mice administered 10^9 vg i.v. showed low levels of plasma enzyme activity only up to 2 months post-treatment. A stark contrast was seen in mice treated with 10^{10} vg for all routes of administration, with high levels of enzyme activity observed in all treated groups. At this dose, plasma IDUA activity in males (Figure 1B) was approximately 10 times higher than that of activity in females (Figure 1C). In males administered 10^{10} vg, IDUA activity in plasma was 1,000–10,000 times higher than normal heterozygote levels, while in most females, activity was 100–1,000 times that of normal levels. While animals treated via IV and IT+IV with 10^{10} vg appeared to have slightly higher plasma activity than IT-treated animals, the difference was not statistically significant. All animals treated at a dose of 10^{10} vg thus showed stable and supraphysiological levels of IDUA activity in plasma throughout the duration of the experiment.

Untreated MPS I mice exhibited high levels of urine GAG excretion compared to normal IDUA heterozygotes. There was no effect on urine GAGs in animals treated with 10^9 vg RGX-111 IV or IT (Figure 1D), nor at the 10^7 and 10^8 vg doses (data not shown). In contrast, animals treated with 10^{10} vg RGX-111 had urine GAG levels that were normalized and stayed at normal levels for the duration of the experiment regardless of the route of administration. MPS I mice administered RGX-111 IT, IV, or IT+IV at a dose of 10^{10} vg (5×10^{11}/kg) thus expressed supraphysiological levels of circulating IDUA activity that normalized GAG levels excreted in the urine as a hallmark of disease.

Tissue IDUA activity following RGX-111 administration

All animals were euthanized between 6 and 9 months post-treatment (see note in materials and methods), tissues collected, and lysates assayed for IDUA activity (Figure 2). IDUA activity was low to undetectable in tissues of animals administered 10^7–10^8 vg AAV-IDUA.

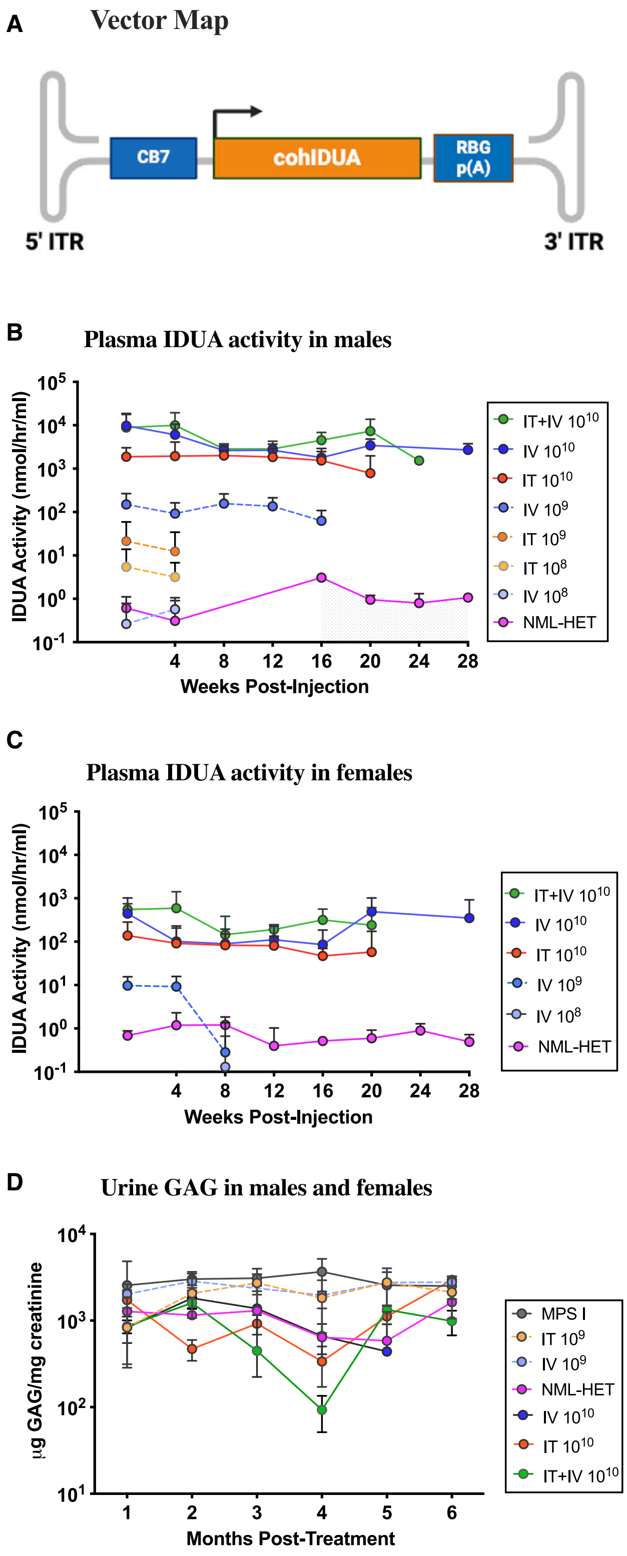

Figure 1. Plasma IDUA activity and urine GAGs in MPS I mice administered RGX-111

(A) Schematic of the AAV9-IDUA (RGX-111) vector. AAV 5′ and 3′ iterative terminal repeats (ITRs) are shown. CB7, cmv immediate-early enhancer, chicken β-actin promoter and intron; cohIDUA, codon-optimized human IDUA coding sequence; RBG p(A), rabbit β-globin polyadenylation signal. (B) Mean plasma IDUA activity in males. (C) Mean plasma IDUA activity in females. (D) Excreted GAGs in urine. Route of administration and dose in vector genomes (vg) are shown in the key for each line. The mean ± SD is shown for each data point, $N = 4–7$ for males and 4–7 for females. GAG levels in untreated MPS I animals were significantly higher than normal controls ($p < 0.0001$) and significantly higher than animals treated with 10^{10} vg RGX-111 ($p < 0.0001$). There was no significant difference between the 3 routes of treatment, nor between treated animals and normal controls. In animals treated with 10^9 vg RGX-111, there was no significant difference between treated animals and untreated MPS I-affected controls.

IDUA activity was low to undetectable in tissues of both male and female mice administered 10^9 vg AAV-IDUA IT (Figure S1A). However, male mice administered 10^9 vg IV had 10 times higher than normal levels of enzyme activity in the liver and normal levels in the spleen, while activity in female mice was low to undetectable (Figure S1B). In contrast, significantly different results were seen in animals administered 10^{10} vg. Data are graphed separately for males (Figure 2A) and females (Figure 2B) due to the substantial differences between these two groups. IDUA activity in treated males exceeded normal by 10-fold in most tissues and by 1,000-fold in liver. Males had 10 times higher levels of IDUA activity than females in the brain, liver, and spleen. All routes of administration showed high and comparable levels of enzyme activity in all tissues except in the brain, where IDUA activity was 10-fold higher after IT administration compared to IV or IT+IV treatment. RGX-111 administered IT, IV, or IT+IV at a dose of 10^{10} vg (5×10^{11}/kg) thus restored or superseded normal IDUA levels in all tissues.

Effect of RGX-111 administration on GAG storage in tissues

Tissues from MPS I animals had high levels of GAGs compared to normal heterozygotes. GAG accumulation in tissues was unaffected by RGX-111 administered at doses of 10^7 or 10^8 vg (data not shown). At a dose of 10^9 vg (Figure 2C), GAGs were normalized only in peripheral tissues (heart, liver, lung, kidney) of IV-administered male mice. However, administration of 10^{10} vg RGX-111 normalized tissue GAG levels in all animals regardless of the treatment route (Figure 2D). The only exception was kidney, where animals treated with 10^{10} vg IT or IV had reduced GAGs that trended toward normal, while animals treated with 10^{10} vg IT+IV had normalized levels of GAGs. RGX-111 administered IT, IV, or IT+IV at a dose of 10^{10} vg (5×10^{11}/kg) thus normalized GAG storage in all tissues.

Vector biodistribution by qPCR

Total DNA extracted from tissue homogenates was assayed for the presence of h*Idua* vector sequences by qPCR (Figure 2E). In IT-treated animals, no vector sequences were detected at the 10^7, 10^8, or 10^9 vg doses, although 2 of the male animals that received 10^9 vg showed a low copy number in the liver (Figure S2A). In IV-treated animals, no vector sequences were detected at the 10^7 or 10^8

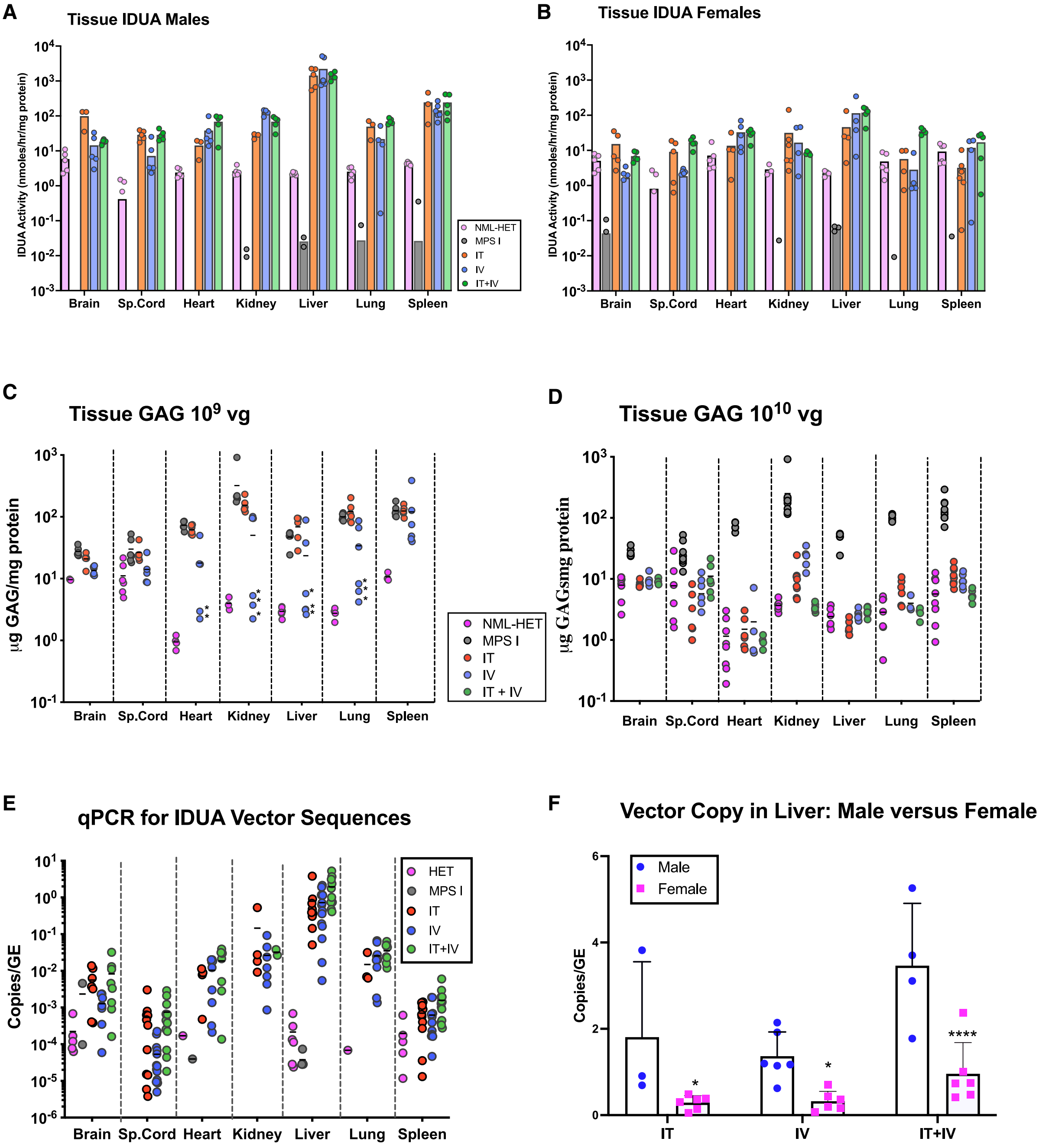

Figure 2. Tissue IDUA activities, GAG accumulation, and vector biodistribution in MPS I mice administered RGX-111

Each data point indicates a value from a single animal, with the mean shown as a horizontal bar. Tissues are indicated across the bottom of each graph. Route of administration or control group are indicated in the key. (A) Tissue IDUA activity in males. (B) Tissue IDUA activity in females. *N* = 3–5 for males and 3–5 for females. *N* values vary since some animals were euthanized and tissues retrieved for histological analysis. (C) Tissue GAGs, 10^9 vg. In animals treated with 10^9 vg RGX-111, GAG levels were normalized only in IV-treated males (marked with an asterisk). (D) Tissue GAGs, 10^{10} vg. GAG levels from animals treated with 10^{10} vg RGX-111 were normalized and similar to normal controls. (E) DNA extracts from tissues (indicated at the bottom) were assayed for the presence of *Idua* sequences by qPCR. The lower limit of detection was determined by analysis of genomic DNA samples collected from heterozygote controls (<0.001 vector copies/genome equivalent [GE]). Not shown are samples from unadministered animals (i.e., untreated MPS I and normal heterozygote controls) giving a negative signal. (F) Vector copy numbers were significantly different in liver DNA extracts from male vs. female animals, *$p < 0.05$ and ****$p < 0.001$.

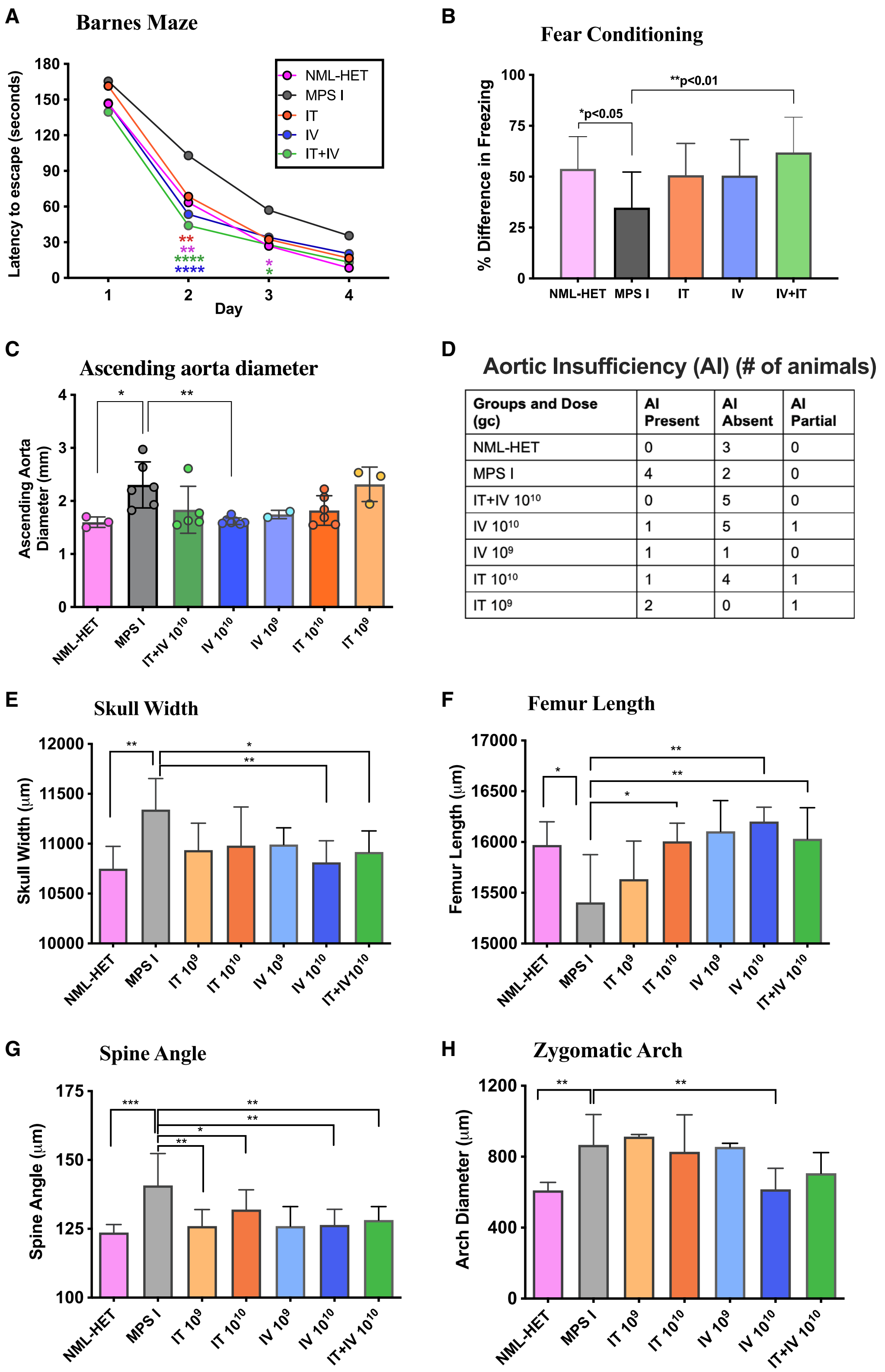

Groups and Dose (gc)	AI Present	AI Absent	AI Partial
NML-HET	0	3	0
MPS I	4	2	0
IT+IV 10^{10}	0	5	0
IV 10^{10}	1	5	1
IV 10^{9}	1	1	0
IT 10^{10}	1	4	1
IT 10^{9}	2	0	1

(legend on next page)

doses, while animals that received 10^9 vg had low copy numbers in the liver and spleen ranging from 0.001 to 0.01 copies per cell (Figure S2B). In contrast, animals administered 10^{10} vg IT+IV showed a high copy number (average of 2 copies/cell) in the liver, while IT- and i.v.-administered animals had an average of 0.3 and 0.2 copies/cell, respectively. In the brain, the IT and IT+IV groups averaged 0.05 and 0.02 copies/cell, respectively, while the IV group was slightly above the limit of detection at 0.002 copies/cell. There was no significant difference in copy numbers among the various routes of administration in the other organs that were assayed. We observed that in the livers of mice treated with 10^{10} vg, male mice had a significantly higher vector frequency than female mice, regardless of the route of injection ($p < 0.05$ for all groups; Figure 2F), and that these higher vector copy numbers were associated with higher levels of enzyme in the livers of male mice. Lower vector frequency in female livers was correlated with lower levels of IDUA enzyme activity in the liver. This sex difference in vector copy number was not observed in other tissues, including the brain. These data demonstrate that (1) liver was a primary site of transduction for IV-, IT-, and IT+IV-administered animals, followed by kidney, heart, and brain, and (2) vector administered IT was released from the IT space into the circulation, leading to transduction of liver and other peripheral tissues (Figure 2E).

Prevention of neurologic decline

MPS I animals treated with 10^9 and 10^{10} vg RGX-111 were evaluated for neurocognitive function at 6 months of age (4 months post-treatment) using the Barnes maze, a test of spatial navigation and memory, and the fear conditioning test, also a test of learning and memory. In the Barnes maze, animals were evaluated in four trials per day for 4 days with a maximum trial time of 180 s. Animals treated with 10^9 vg either IT or IV showed no significant improvement in time to escape compared to untreated MPS I animals (Figure S3A). In contrast, animals treated with 10^{10} vg RGX-111 showed a significant improvement in time to escape, regardless of treatment route, locating the escape hole in 65 s or less by day 2, while untreated MPS I mice showed an escape time of about 100 s (Figure 3A). Locating the escape hole was progressively faster each day for the AAV-treated animals compared to untreated MPS I controls. Time to escape on day 2 was not significantly different between animals administered 10^{10} vg RGX-111 (all routes of administration) and normal heterozygote control animals.

The fear conditioning test measures the fear response, assessed as freezing time, to a cue presented prior to a mild foot shock during training trials. MPS I animals showed reduced freezing responses when presented with the aversive cue compared to normal heterozygote controls (Figure 3B). Animals treated with 10^9 vg RGX-111 either IT or IV failed to show any increase in freezing response compared to untreated MPS I animals (Figure S3B). In contrast, MPS I mice treated IT+IV with 10^{10} vg RGX-111 demonstrated a freezing time significantly improved compared to untreated MPS I mice (Figure 3B) and not significantly different from normal heterozygote controls. IV- and IT-treated animals showed freezing times that were not significantly different from IT+IV-treated animals. Treatment with RGX-111 at a dose of 10^{10} vg (5×10^{11}/kg) thus prevented cognitive decline in MPS I mice regardless of the route of vector administration.

Prevention of cardiac dysfunction

Male MPS I mice exhibit significant cardiac dysfunction in the form of ascending aortic dilation, aortic insufficiency (AI), and decreased left ventricular function.[48–50] For this study, test animals were analyzed by high-resolution ultrasound biomicroscopy at 6 months of age (4 months post-treatment) to assess the effect of RGX-111 administration on cardiac valve function (Figures 3C and 3D). The mean inner ascending aortic diameter in normal mice was 1.7 mm and was significantly increased in MPS I mice to 2.3 mm (Figure 3C). Compared to untreated mice, ascending aortic diameter was reduced in MPS I animals treated with 10^{10} vg RGX-111 IT (1.8 mm), IV (1.6 mm, significant), and IT+IV (1.8 mm) and approached normal wild-type values. Animals administered 10^9 vg IT did not show a significant difference in diameter (2.3 mm) compared to untreated MPS I animals (2.3 mm), while animals administered 10^9 vg IV had a decreased aortic diameter (1.8 mm) compared to MPS I animals, although not significantly so (Figure 3C). AI was observed in 67% of male MPS I animals, while age-matched normal heterozygotes showed no AI (Figure 3D). In contrast, MPS I mice showed 16%, 14%, and no AI after 10^{10} vg RGX-111 infusion IT, IV, and IT+IV, respectively. In 10^9 vg IT-treated animals, 2 out of 3 animals had AI, while in 10^9 vg IV-treated animals, 1 had AI, and the other did not (Figure 3D). All animals treated at the 10^{10} vg dose (5×10^{11} per kg) were thus significantly improved or trended toward improvement in cardiac function compared to untreated MPS I mice, with no significant difference between treatment groups.

Prevention of skeletal disease

One week before euthanasia, all mice were imaged by computed tomography (CT), and measurements were made for skull width, femur length, spine angle, and zygomatic arch, each showing a

Figure 3. Effect of RGX-111 treatment on neurocognition, cardiac function, and skeletal dysplasias

Route of administration or control group are shown in the key. (A) Barnes maze. The Barnes maze was used to assess the effect of vector administration on spatial learning and memory. Testing was carried out in four trials per day over 4 days for all groups (N = 13–16). $^{**}p < 0.01$ and $^{***}p < 0.0001$. Mean latency to escape for the 4 trials is shown for each day. (B) Fear conditioning. All groups were evaluated for fear-conditioned response. Mean freezing time as a cued response is shown (N = 13–16). $^{*}p < 0.05$ and $^{**}p < 0.01$. (C) Cardiac ultrasound measurements of ascending aorta diameter in mm. Each bar represents the mean of individual values (dots) ± SD error bars. (D) Doppler interrogations of aortic valve for valve insufficiency (NML-HETN = 3; MPS I N = 6; 10^9 vg N = 3; 10^{10} vg N = 6). Skeletal outcomes were determined by micro-CT analysis of control groups and animals treated with 10^9 or 10^{10} vg RGX-111 via the route of administration indicated at the bottom of each graph (NML-HET N = 5–6; MPS I N = 4; 10^9 vg N = 3; 10^{10} vg N = 6). All skeletal outcomes shown are in male mice. (E) Skull width. (F) Femur length. (G) Spine angle. (H) Zygomatic arch. $^{*}p < 0.05$ and $^{**}p < 0.01$. Absence of bracket indicates no significant difference between groups.

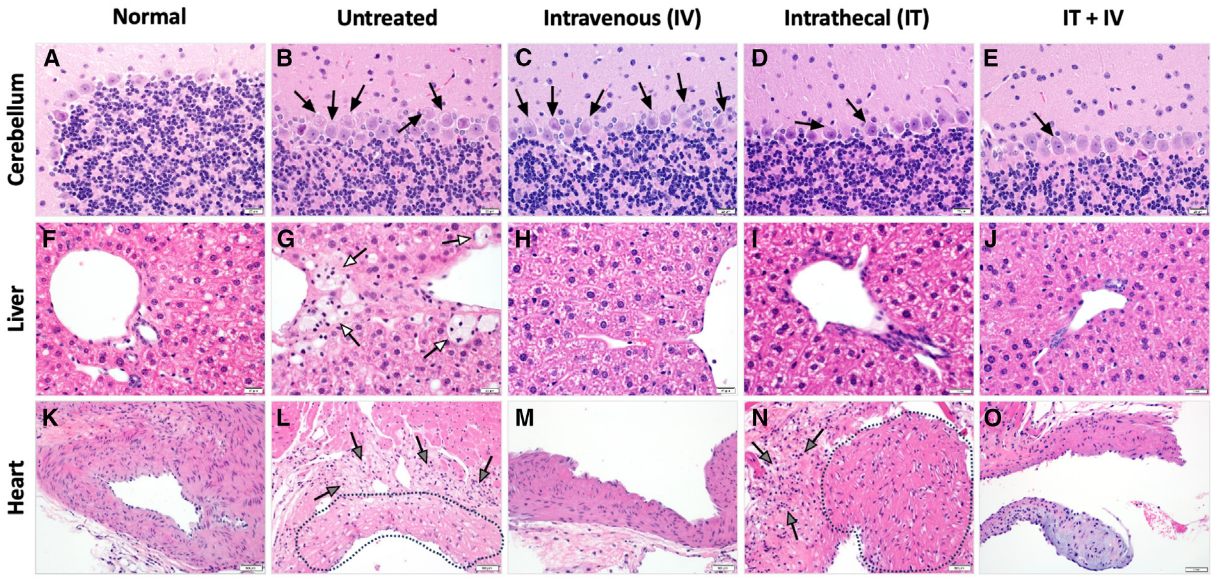

Figure 4. Prevention of cerebellar, hepatic, and cardiovascular pathology in treated MPS I mice

H&E composite. (A, F, K) sections from normal tissues. In the cerebellum, liver, and aortic outflow tract of the untreated mice, note the intraneuronal Purkinje cell vacuolization (B, black arrows), foam cell accumulation (G, white arrows), and extensive vacuolization (L, encircled by the dashed black line) of the tunica media of large extra-cardiac vessels and infiltration of inflammatory cells (L, gray arrows). In the cerebellum, there was a progressive decline in the severity of Purkinje cell vacuolization from the IV- (C, black arrows) to the IT- (D, black arrows) to the IT+IV-treated (E, black arrows) mice. In the liver, vacuolated foam cells were not observed in the IV- (H), IT- (I), or IT+IV-treated (J) mice. In the vascular outflow tract, there was a marked reduction in vacuolization and inflammatory cell infiltration in the IV-treated mice (M) with persistent but less severe findings in the IT (N, gray arrows and encircled black dashed line) and IT+IV-treated (O, encircled black dashed line) mice. Scale bars: (A–E) 20 μm, (F–J) 20 μm, and (K–O) 50 μm.

significant difference between affected and unaffected controls. MPS I female mice did not show a deficiency in skeletal outcomes when compared to normal heterozygotes, so data are shown for male animals only (Figures 3E–3H). In males treated with 10^{10} vg RGX-111, both IV- and IT+IV-administered animals showed a significant difference in skull width compared to untreated MPS I mice and were not significantly different from normal males (Figure 3E). IT-treated male mice trended toward normalization, although the difference between those and untreated MPS I mice was not significant. The 10^9 vg dose had no effect on skull width regardless of route of administration. Femur length was normalized in all 10^{10} vg-treated males (Figure 3F) irrespective of route of vector administration. At the 10^9 vg dose, an improvement of femur length was seen in both IT- and IV-treated animals. Spine angle was also normalized in all male mice treated at the 10^9 vg dose regardless of the route of administration, while at the 10^{10} vg dose, IV- and IT+IV-treated mice showed normalization. The 10^{10} vg IT-treated mice trended toward normal but were not significantly different from MPS I mice nor the 10^9 vg-treated mice (Figure 3G). Zygomatic arch trended toward normal in males administered 10^{10} vg RGX-111 IT and IT+IV, while IV-treated male mice were significantly normalized compared to MPS I mice (Figure 3H). RGX-111 delivered at a dose of 10^{10} vg (5×10^{11} vg/kg) IT, IV, or IT+IV thus prevented the emergence of skeletal abnormalities in MPS I mice.

Histology

Tissues from mice treated with 10^{10} vg RGX-111 were evaluated by H&E (Figure 4), Alcian blue staining for GAG storage accumulation (Figure 5), and immunohistochemistry (IHC) for IDUA using an antibody specific for hIDUA (Figure 6). In contrast to normal mice, untreated MPS I mice had extensive, focal to multifocal, intraneuronal vacuolization in cerebellar Purkinje cells (Figure 4B), with progressively decreasing vacuolization observed in the IV- and IT-treated mice and only rare cerebellar vacuolization observed in the combination (IT+IV)-treated mice (Figures 4C–4E). There was a significant accumulation of vacuolated foam cells within the centrolobular region of the liver in untreated mice (Figure 4G), with no vacuolated cells observed in any of the 3 treatment groups (Figures 4H–4J). Similarly, in the heart, there was extensive vacuolization of the tunica media of large extra-cardiac vessels with infiltration of vacuolated foam cells in the untreated mice (Figure 4L). Vascular pathology was not identified in the IV-treated mice (Figure 4M), and there was decreased tunica media vacuolization and inflammatory infiltration in the IT- (Figure 4N) and IT+IV-treated mice (Figure 4O). These vacuoles in untreated mice correspond to the accumulation of Alcian blue-positive material in all tissues (Figures 5B–5G and 5L). Alcian blue-positive material was not observed in the cerebellum for any of the 3 treatment groups (Figures 5C–5E). In the liver, no Alcian blue staining was observed in IV or IT+IV groups (Figures 5H

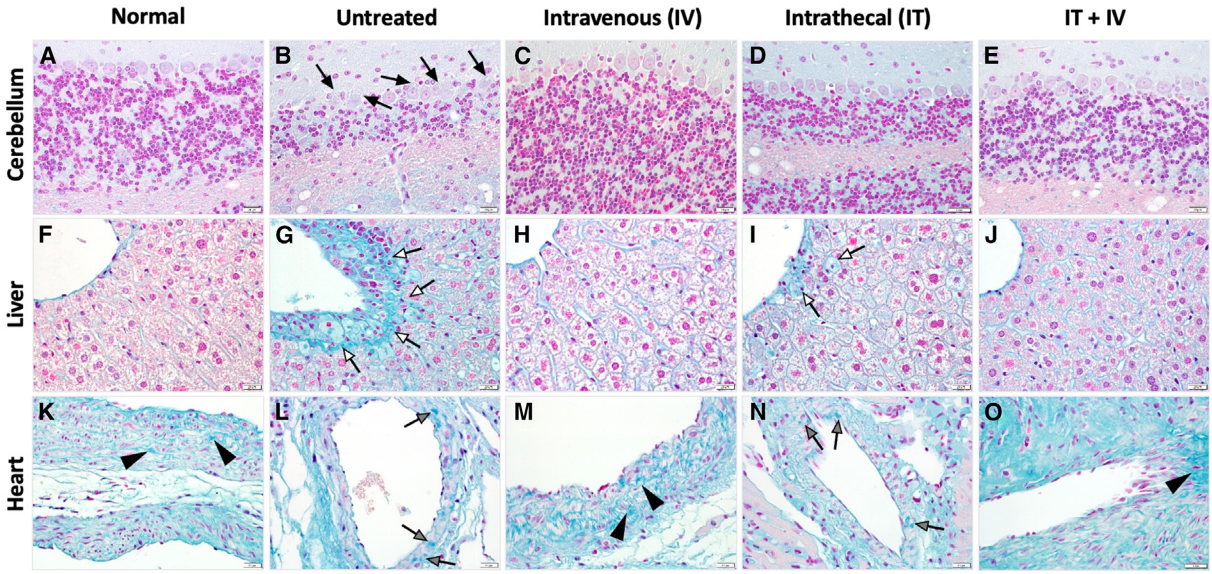

Figure 5. Prevention of polysaccharide accumulation in the cerebellum, liver, and heart of treated MPS I mice

Alcian blue composite. (A, F, K) sections from normal tissues. In the cerebellum, liver, and aortic outflow tract of the untreated mice, note the accumulation of Alcian blue-positive material within the Purkinje cells (B, black arrows), perivascular foam cells (G, white arrows), and as irregularly sized amorphous aggregates in the arterial wall (L, gray arrows). In the cerebellum, Alcian blue-positive material was not see in the Purkinje cells from the IV- (C), IT- (D), or IT+IV-treated (E) mice. In the liver, Alcian blue staining material was not seen in the IV- (H) or IT+IV-treated (J) mice, while decreased amounts were observed in the IT-treated mice (I). In the vascular outflow tract, there was a small amount of granular, dispersed Alcian blue-positive material in the IV- (M, black arrowheads) and IT+IV-treated (O, black arrowheads) mice, which was similar to the normal control (K). Fewer and small amorphous aggregates of Alcian blue-positive material were seen in the IT-treated mice (N, gray arrows). Scale bars: (A–E) 20 μm, (F–J) 20 μm, and (K–O) 20 μm.

and 5J), while the IT-treated group showed a low level of Alcian blue staining (Figure 5I). In the heart, in contrast to the finely granular Alcian blue-positive material within the arterial walls of normal mice (Figure 5K), there was an accumulation of irregularly sized and amorphous aggregates of polysaccharide within the arterial wall of untreated mice (Figure 5L). These amorphous aggregates were not seen in the vascular walls of the IV- (Figure 5M) and IT+IV-treated (Figure 5O) mice, while fewer and smaller aggregates were seen in the vascular walls of the IT-treated (Figure 5N) mice. In contrast to normal (Figures 6A–6F and 6K) and untreated (Figures 6B–6G and 6L) mice and regardless of tissue, staining for IDUA was detected in all treated mice. Within the brain, intraneuronal immunostaining for IDUA was sporadic (Figures 6C–6E), with IT-treated animals demonstrating the highest density of immunopositive cells, followed by IT+IV-treated mice, with lesser numbers of IDUA-positive cells in the i.v.-treated mice. Within the liver, IDUA-positive hepatocytes and sinusoidal lining cells were identified in the IV- (Figure 6H), IT- (Figure 6I) and IT+IV-treated (Figure 6J) mice, with the highest density of IDUA-positive cells seen in IV-treated mice. Within the heart, IDUA-positive stromal cells and cardiomyocytes were identified in the IV- (Figure 6M), IT- (Figure 6N), and IT+IV-treated (Figure 6O) mice. A notable difference in the density of IDUA-positive cells was observed in the liver between male and female mice, with a significantly higher proportion of IDUA-positive cells observed in male mice for all routes of treatment (Figure 7). However, no significant difference in IDUA staining in the brain was observed between males and females (Figure S4). Overall, these results provide histologic evidence for substantial *in vivo* transduction of liver, brain, and heart in animals administered 10^{10} vg (5×10^{11} vg/kg) RGX-111 IT, IV, or IT+IV with normalized levels of GAG.

DISCUSSION

In this study, we compared different routes and doses of RGX-111 administration and their effect on multisystem disease manifestations in MPS I mice. We found that a dose of 10^{10} vg was sufficient to restore IDUA enzyme activity and normalize GAG levels in urine and tissues as assessed biochemically and histologically. Lower doses of RGX-111 (10^9, 10^8, and 10^7 vg) did not consistently restore IDUA enzyme activity, particularly in female mice, thus defining 10^{10} vg (5×10^{11} vg/kg) as the minimal effective dose. Yet this modest and minimal effective dose was sufficient to prevent the emergence of biochemical, cardiac, neurological, and skeletal manifestations of disease when delivered by the IT, IV, or IT+IV route of administration. Systemically administered vector thus delivered sufficient enzyme to the CNS to allow retention of neurologic function as assessed by Barnes maze and fear conditioning behavioral analyses. While IT infusion into the CSF effectively delivered vector to the CNS, there was also substantial vector release and systemic redistribution resulting in transduction of the liver, secretion of enzyme into the circulation, and prevention of peripheral cardiac and skeletal manifestations of disease.

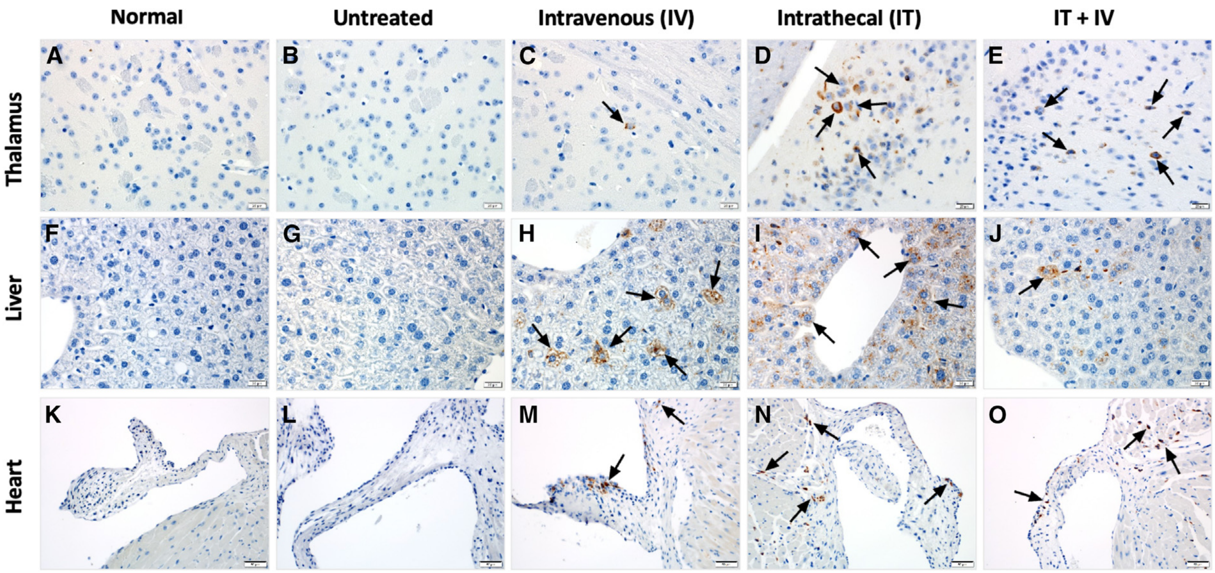

Figure 6. Detection of IDUA-positive cells in treated MPS I mice

IDUA composite. (A, F, K) sections from normal tissues. In contrast to the thalamus (B), liver (G), and aortic outflow tract (L) from the untreated mice, note the IDUA-positive cells (black arrows) in the IV- (C, H, and M), IT- (D, I, and N) and IT+IV-treated (E, J, and O) mice. In the thalamus, the highest density of IDUA-positive cells was seen in the IT-treated mice (D, black arrows), while in the liver, the highest density was seen in the IV-treated mice (H). As described in materials and methods, the IDUA antibody used is specific for human IDUA and does not recognize mouse IDUA in normal mice. Scale bars: (A–E) 20 μm, (F–J) 20 μm, and (K–O) 50 μm.

A significant 10-fold higher level of IDUA enzyme activity was observed in males compared to females, primarily in the plasma, brain, and liver. IDUA activity levels also trended lower for females in other organs, although the difference was not significant. In general, the level of enzyme in the different organs in different animals corresponded to the level of enzyme in the liver and the plasma. GAG levels were normalized in both males and females administered 10^{10} vg AAV9-IDUA. Higher efficiency of AAV9 gene transfer into the liver in male mice has been documented previously and appears to be testosterone dependent.[51–54] A systematic evaluation of the influence of sex on transduction by recombinant AAV2 (rAAV2) indicated that transgene expression after liver-targeted delivery of vector particles was between 5- to 13-fold higher in male mice compared with female mice, irrespective of the promoter, cDNA, or mouse strain.[51] Molecular analysis revealed that the rAAV genome was stably retained in male liver at levels that were 7-fold higher than those observed in females. Maguire et al. showed a sex difference in mouse strains infused systemically with an AAV9 encoding firefly luciferase.[53] AAV9 gene transfer in the brain after vascular delivery in adult GM1 mice was found to be more efficient in females than males,[52] which could be partly due to lower affinity for liver. This suggests that liver tropism/affinity may be a major factor in determining the bioavailability of AAV9 to transduce other organs after systemic delivery. This gender effect on AAV liver transduction has not been reported for other mammalian species such as non-human primates. While there is clearly a preference for higher activity in livers of males than females, we do not see increased IDUA activity in the brains of female mice compared to male mice (Figure S4).

A key goal of genetic therapy for MPS I and MPS diseases in general is to provide a remedy for neurologic manifestations of disease.[11] This was readily accomplished by direct (IT) *Idua* gene transfer into the CSF for expression of the missing enzyme, verified histologically by IHC (Figure 6D). IV administration of 10^{10} vg AAV9-IDUA also restored brain IDUA activity, normalized brain GAGs, and prevented neurocognitive decline in MPS I mice. AAV9 has been demonstrated to transduce CNS tissues after IV administration,[42] so in our study, brain IDUA could also be generated by expression of vg delivered to the CNS. However, histologically we observed only a low frequency of transduced IDUA-positive cells in the CNS after IV vector administration (Figure 6C). Therefore, the high levels of circulating IDUA that we observed provide a more likely source of enzyme for distribution to CNS tissues in this study. In support of this argument, high doses of aldurazyme (20× normal) have been shown to prevent neurologic decline in MPS I mice,[55] and liver-specific transgene expression has been shown to reduce GAGs in the brain and prevent development of cognitive dysfunction in MPS I and MPS II mice.[31,56]

A substantial amount of IT-administered AAV9 was released from the CSF and redistributed into the circulation. As a result, the highest vector distribution and IDUA activity were found in the liver for both IV- and IT-administered animals. Comparing outcomes in males vs. females administered 10^9 vg AAV9-IDUA IV and in males administered 10^9 vg AAV9-IDUA vector IV vs. IT provides insight into the relationship between transduction frequency and relative effectiveness. 10^9 vg AAV9-IDUA administered IV to male mice was sufficient to restore enzyme activity, normalize GAGs, and benefit the animal's neurocognition. However, in males administered

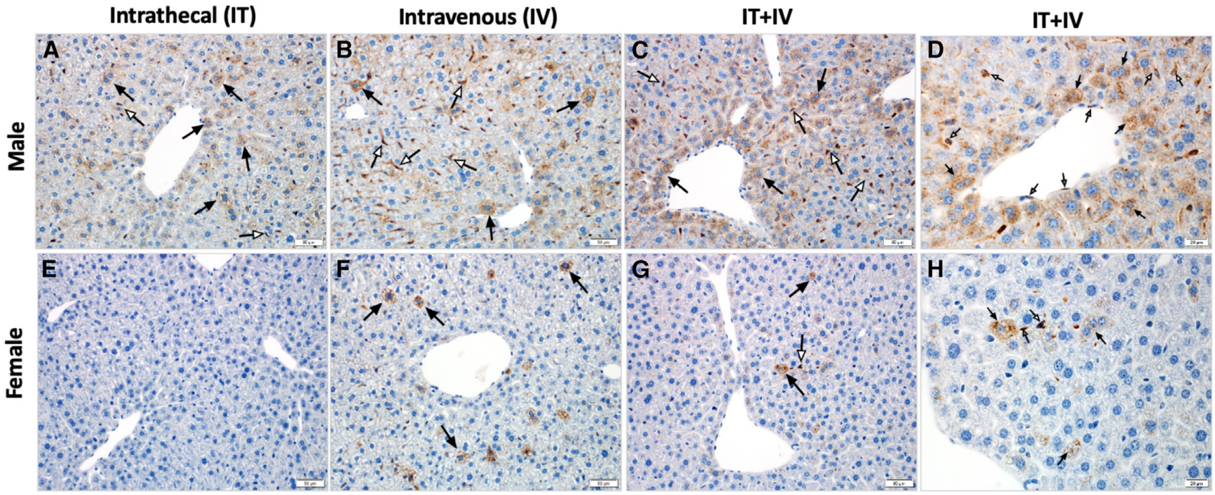

Figure 7. Higher density of IDUA-positive cells in treated male mice vs. female mice

In contrast to the absent to low number of IDUA-positive hepatocytes (black arrows) and Kupfer cells (white arrows) in the RGX-111-treated (10^{10} vg) female mice (E–H), note the increased density of IDUA-positive cells (black and white arrows) in the treated male mice, irrespective of injection site (A–D). Shown in higher magnification in (D) and (H), the male IT+IV-treated mice (D) showed an increased density of IDUA-positive hepatocytes (black arrows), Kupfer cells (white arrows), and endothelial cells (gray arrows) as compared to the IT+IV-treated female mice (H). Scale bars: (A–C and E–G) 50 μm and (D and H) 20 μm.

10^9 vg AAV9-IDUA IT or in females given 10^9 vg AAV9-IDUA, the resulting *in vivo* transduction was insufficient to provide sustained IDUA expression and normalization of GAGs. A dose of 10^9 vg administered IV in males thus overcomes a pharmacokinetic threshold to achieve sufficient transduction that metabolically and physiologically remedies manifestations of MPS I. Male and female mice administered lower doses of 10^7 or 10^8 vg AAV9-IDUA IV or IT showed little or no enzyme activity, no GAG reduction, and little to no detectable copies in the tissues analyzed. A dose of 10^{10} vg (5×10^{11} per kg) was thus assessed as minimally effective for either route of administration and for both male and female mice.

A wide range of vector doses have been employed in the clinical application of AAV,[57] with some highly effective products such as Zolgensma administered at a dose of 10^{14} vg/kg.[58,59] However, AAV vectors can be toxic when administered at such high doses, with fatalities reported that were associated with liver failure and coagulopathies.[60] Clinical studies in the development of gene therapy for hemophilia B demonstrated that AAV administered systemically at high doses can elicit an anti-capsid T cell response causing liver damage and shutting down therapeutic transgene expression.[61] George et al.[62] demonstrated that the risk of such T cell responsiveness can be mitigated through systematic dose reduction, reaching a final safe dose of 5×10^{11} vg/kg.

The results reported in this paper are thus highly supportive of AAV9-IDUA, RGX-111, as a genetic therapy for MPS I. Recognizing the potential necessity of dose scale-up for human application, our minimally effective dose of 5×10^{11} vg/kg is in the range that others have found in clinical studies to minimize the risk of an adverse T cell immune response.[62] At the same time, this dose was sufficient to metabolically restore the recipient animal and normalize GAG storage levels. There was effective transduction of CNS tissue after CSF-directed IT vector infusion but also substantial vector release with transduction of the liver and circulation of enzymes for metabolic cross-correction. At the same time, IV AAV9-IDUA administration brought about high levels of IDUA in the circulation, delivering enzymes to all tissues, including the brain. Both IT and IV routes of administration prevented the emergence cardiac, skeletal, and neurologic manifestations. Overall, these results predict effective outcomes for CSF-directed RGX-111 (ClinicalTrials.gov: NCT03580083) with respect to both neurologic and peripheral (cardiac and skeletal) manifestations. The results also suggest effective outcomes for IV-administered RGX-111 in addressing neurologic as well as peripheral manifestations of MPS I. RGX-111, AAV9-IDUA, administered either IV or through the CSF, thus has the potential to address the unmet need for increased delivery of IDUA enzyme to the brain and other tissues as improved therapy for MPS I.

MATERIALS AND METHODS

AAV vector assembly and packaging

The CB7-regulated, codon-optimized hIDUA construct (Figure 1A) was designed, assembled, and packaged into AAV9 virions at REGENXBIO (RGX-111). Procedures for vector packaging, purification, and titering have been previously described.[63]

Animal care

The C57BL/6 IDUA-deficient mouse strain (MGI:2651471) was kindly provided by Dr. Elizabeth Neufeld. Animals were bred and maintained under specific pathogen-free conditions and provided food and water *ad libitum*. Mouse care and experimental procedures were conducted under University of Minnesota Institutional Animal Care and Use

Committee (IACUC) approval. Experimental and control animals were produced by breeding *IDUA*$^{-/-}$ males with *IDUA*$^{-/-}$ or *IDUA*$^{+/-}$ females. Mice elicit a substantial immune response against hIDUA, so we initially attempted to immunotolerize MPS I animals with Aldurazyme (laronidase). However, this approach failed to prevent an immune response, likely because a lower dose and duration of Aldurazyme administration was used in comparison to what we previously reported.[20] IDUA activity in Aldurazyme-administered animals was extinguished by 2–4 weeks post-AAV9-IDUA treatment, which was confirmed to be due to an anti-IDUA antibody response by ELISA (data not shown). For the experiments described in this study, immune response was thus suppressed by administration of cyclophosphamide (CP, Sigma-Aldrich, St. Louis, MO) weekly at a dose of 120 mg per kg intraperitoneally starting on day 3 after vector infusion. After 2 months of weekly CP administration, the drug was administered every other week for the duration of the study. All animals in all groups were immunosuppressed with CP, including untreated MPS I-affected controls as well as normal unaffected controls. Animals were monitored for weight loss during CP administration, and CP was withdrawn if animals lost more than 10% of their body weight. In a few (5) female animals, CP administration did not prevent rapid loss of IDUA enzyme activity, likely due to an immune response, so these animals were removed from the study.

Routes of vector infusion

RGX-111 was administered to 2-month-old *IDUA*$^{-/-}$ animals via IT, IV, or IT+i.v. routes. Doses ranged from 10^7 to 10^{10} vg. For the 10^{10} vg dose, N = 9–12 animals (6 males, 3–6 females) were included for each treatment group, while N = 6 (3 males, 3 females) animals were injected with 10^7, 10^8, or 10^9 vg. For IT injections, animals were administered a 10 μL RGX-111 vector as described previously[20] at the specified dose in the lumbar region, between L5 and L6. Animals injected IV received a volume of 200 μL RGX-111. For the IT+IV route, animals received the vector IT (10^{10}vg/10 μL), followed by IV administration (10^{10} vg/200 μL).

Plasma and tissue lysate preparation

Blood was collected monthly by submandibular puncture into heparinized tubes, processed for plasma, and stored at −20°C until assayed. The experimental study design required animals to be euthanized 6 months post-AAV injection (8 months of age). However, due to quarantine requirements during the COVID pandemic, euthanasia dates for the various groups had to be adjusted (all times post-AAV treatment): IT-treated animals, 6–9 months; IV-treated animals, 6–8 months; and IT+IV-treated animals: 6–9 months. At euthanasia, animals were transcardially perfused with 0.9% sodium chloride. Organs were harvested and frozen at −80°C until assayed. Organs were homogenized using a Bullet blender STORM bead homogenizer (Next Advance) and clarified by centrifugation. Tissue lysates were stored at −80°C until assayed.

Enzyme assays

The IDUA assay was carried out as previously described.[64] Briefly, activity was determined fluorometrically using 4-methylumbellferyl α-L-iduronide as substrate (4MU-iduronide; Glycosynth) in 0.4 M sodium formate buffer (pH 3.5) at 37°C for 1 h. 0.2 M glycine carbonate buffer (pH 10) was added to stop the reaction. Fluorescence was measured at 365 nm excitation and 460 nm emission using a BioTek plate reader. IDUA enzyme activity is expressed as nmol/h/mL for plasma and nmol/h/mg protein for tissue extracts. Protein in tissue samples was determined using the Pierce assay.

Glycosaminoglycan assay

Urine was collected monthly and frozen at −20°C until assayed for GAG content. Urine and tissue GAG assays were carried out as previously described.[63] Tissue lysates from liver, spinal cord, and brain were incubated with proteinase K, DNaseI, and RNase overnight at 55°C. GAG contents were measured using the Blyscan Sulfated GAG Assay kit as per the manufacturer's instructions (Biocolor Life Science Assays; Accurate Chemical, NY). Creatinine was assayed according to the manufacturer's instructions (Sigma-Aldrich, no. MAK080). Tissue GAG levels were normalized to protein and are expressed as μg GAG/mg protein. Urine GAG levels are expressed as μg GAG/mg creatinine.

Quantitative polymerase chain reaction

DNA was extracted from tissue homogenates using the 5 PRIME Archive Pure DNA Purification kit according to manufacturer's instructions. For *Idua* qPCR, 25 μL reactions contained 60 ng of DNA template, 2× IQ SYBR Green Supermix (Bio-Rad), 5 pmol/μL of forward primer (5-GGACATCATGAAGCCCCTT-3'), and 5 pmol/μL of reverse primer (5'-AAAAGTAACGTTATCACACAACCT-3'). PCR conditions were 95°C for 10 min, followed by 40 cycles of 95°C for 15 s and 60°C for 1 min in a Bio-Rad C1000 Touch Thermo Cycler. Serial dilutions of REGENXBIO plasmid RGX-111 were used to generate a standard curve and results are expressed as vector copies per genome equivalent.

Barnes maze

At 4 months post-AAV treatment (6 months of age), animals in all groups were analyzed for memory and spatial navigation using the Barnes maze as previously described.[23,65] Briefly, animals were released in the center of a 20 hole circular maze and given 3 min to explore and find the single hole open to an escape box under the maze. If the mouse did not find the escape box, then it was guided there and left in the escape box for 30 s before being returned to its home cage. The time taken to enter the escape box was recorded as latency to escape. Animals were given four trials per day for 4 days.

Fear conditioning

Animals were evaluated using the fear conditioning test a week after Barnes maze. This associative learning and memory test measures a fear response (i.e., time spent freezing) to a conditioned stimulus (cue) that predicts an unconditioned stimulus (mild foot shock) presented during training trials. Data collection and analysis are semi-automated via a video-monitoring fear-conditioning apparatus (Med Associates). On the conditioning day (training day; day 1), the test chamber was sprayed with a solution of Simple Green as an

olfactory cue, and mice were exposed to a series (five pairings; 60 s intertrial interval) of cue (80 dB white noise tone and light) presentations (15 s in duration) co-terminating with a mild foot shock (0.7 mA, 1 s in duration). 24 h later, contextual fear conditioning was carried out in a test chamber sprayed with Simple Green. Mice were placed in the chamber for 3 min with no sound or light cues and no foot shock. Freezing response was assessed for 3 min. 1–2 h following the contextual test, cued fear testing was conducted in a test chamber with altered contextual elements (white floor and walls with vanilla odor) and consisted of a 3 min baseline (nonspecific freezing behavior) and a 3 min light and sound cue exposure (cued fear) period. The percentage of freezing time was assessed during all sessions.

Echocardiography

Following behavior testing, the male mice were evaluated for cardiac function.[48–50] Cardiac ultrasounds were performed on the Vevo 2100 (VisualSonics, Fujifilm, Toronto, Ontario) using the MS400 probe, an 18–38 MHz probe with lateral resolution of 50–110 μm and an acquisition speed of up to 1,000 frames/s for M-mode studies. Mice were anesthetized with 2% isoflurane and transferred to a heated examination platform. Fur was removed, and skin was cleansed with depilatory and warm water. Temperature, heart rate, and respiratory rate were continuously monitored throughout the study. Heart rates >400 beats/min and temperatures >35°C were maintained during the study. Aortic root and ascending aortic dimensions were measured in triplicate by B-mode imaging. The aortic valve and descending thoracic aorta were evaluated by pulse-wave and color Doppler for the presence or absence of AI and aortic flow reversal (a sign of severe AI). Once the procedure was completed, mice were returned to their cage following recovery from anesthesia.

Computed tomography

At 1 week prior to euthanasia, all experimental animals were anesthetized with 5% isoflurane and underwent micro-CT imaging using a Siemens Inveon PET/CT machine. DICOM files were converted to an Imaris image file using Imaris File Converter software, which was then analyzed using Imaris 3D Analysis software. Micro-CT measurements included zygomatic arch diameter, skull width, femur length, and spine angle.

Histology

Tissues were fixed in 10% neutral buffered formalin, transferred to 70% ethanol, processed into paraffin blocks, and 4 μm sections cut and stained with H&E. For IHC, paraffin-embedded tissue sections were deparaffinized and rehydrated, followed by antigen retrieval using sodium citrate buffer. After quenching endogenous peroxidase and application of a protein block (Dako), IHC was performed by incubating with a sheep anti-IDUA (specific for hIDUA; R&D Systems, Minneapolis, MN; 1:500) overnight at 4°C followed by a biotinylated anti-sheep secondary antibody (Vector Labs, cat# BA6000) and a tertiary LSAB detection reagent (Dako). Diaminobenzidine was used as the chromogen, and Mayer's hematoxylin (Dako) was used as the counterstain. Primary antibodies were substituted with appropriate negative control immunoglobulin (Ig)G for negative control slides. For Alcian blue staining, 4 μm deparaffinized and rehydrated sections were stained with the Alcian blue 1%, pH 2.5 kit (Newcomer, #9102A). Equal numbers of male and female mice were analyzed histologically and are represented in Figures 4, 5, 6, and 7.

Statistical analysis

GraphPad Prism (GraphPad software) was used for all statistical analyses. For IDUA plasma activities and Barnes maze/fear conditioning, data were compared to normal heterozygote levels and untreated MPS I mice using two-way ANOVA followed by Dunnett's multiple comparisons test. Tissue IDUA activity and GAG levels were compared to heterozygote levels using the Kruskal-Wallis test. Echocardiograph and micro-CT data were analyzed using Tukey's multiple comparisons test and Fisher's least significant difference (LSD), respectively. p values <0.05 were considered significant.

DATA AND CODE AVAILABILITY

The data generated during the study are presented in this original research article. Protocols or other information can be requested by contacting the corresponding author.

ACKNOWLEDGMENTS

Behavioral studies were performed in the Mouse Behavior Core at the University of Minnesota (supported by NIH grant NS062158). We thank core director Dr. Lind for assistance with the neurobehavioral testing. This study has been published in abstract form in conference proceedings. This study was funded by REGENXBIO.

AUTHOR CONTRIBUTIONS

L.R.B.: experimental design and coordination, tissue processing, biochemical assays (IDUA activity, GAG, DNA extraction, and qPCR assays), neurobehavioral testing, data compilation and analysis, and lead role in manuscript writing; A.K.H.: IDUA enzyme assay, GAG assay, DNA extraction, and qPCR; H.M.: IDUA enzyme assay, GAG assay, DNA extraction, and qPCR; M.R., IDUA enzyme assay, GAG assay, DNA extraction, and qPCR; M.C.S., study design and data interpretation; A.D.K., animal husbandry; K.F.K. and C.A.F., IT injections; L.O. and C.B.W., IDUA ELISA; E.B., echocardiography and analysis; J.F. and T.C.L., micro-CT radiography and analysis; D.S., histopathologic analysis and manuscript writing; K.H.K., coordination and manuscript writing; N.B., study design and coordination and project management; R.S.M., study design and coordination, project management, data interpretation, and manuscript writing.

DECLARATION OF INTERESTS

The work detailed in this manuscript was sponsored by REGENXBIO, Inc. K.H.K. and N.B. are current or former employees of REGENXBIO, Inc. L.R.B., T.C.L., C.A.F., N.B., and R.S.M. are co-inventors on patents related to the contents of this manuscript. R.S.M. is also an employee of Immusoft Corp.

SUPPLEMENTAL INFORMATION

Supplemental information can be found online at https://doi.org/10.1016/j.omtm.2024.101369.

REFERENCES

1. Neufeld, E.F., and Muenzer, J. (2019). The Mucopolysaccharidoses. In The Online Metabolic and Molecular Bases of Inherited Disease, D.L. Valle, S. Antonarakis, A. Ballabio, A.L. Beaudet, and G.A. Mitchell, eds. (McGraw Hill).

2. Wraith, J.E., and Jones, S. (2014). Mucopolysaccharidosis type I. Pediatr. Endocrinol. Rev. *12*, 102–106.

3. Kubaski, F., de Oliveira Poswar, F., Michelin-Tirelli, K., Matte, U.D.S., Horovitz, D.D., Barth, A.L., Baldo, G., Vairo, F., and Giugliani, R. (2020). Mucopolysaccharidosis Type I. Diagnostics *10*, 161.

4. Cleary, M.A., and Wraith, J.E. (1995). The presenting features of mucopolysaccharidosis type IH (Hurler syndrome). Acta Paediatr. *84*, 337–339.

5. Parini, R., Deodato, F., Di Rocco, M., Lanino, E., Locatelli, F., Messina, C., Rovelli, A., and Scarpa, M. (2017). Open issues in Mucopolysaccharidosis type I-Hurler. Orphanet J. Rare Dis. *12*, 112.

6. Hampe, C.S., Eisengart, J.B., Lund, T.C., Orchard, P.J., Swietlicka, M., Wesley, J., and McIvor, R.S. (2020). Mucopolysaccharidosis Type I: A Review of the Natural History and Molecular Pathology. Cells *9*, 1838.

7. Jameson, E., Jones, S., and Remmington, T. (2019). Enzyme replacement therapy with laronidase (Aldurazyme®) for treating mucopolysaccharidosis type I. Cochrane Database Syst. Rev. *6*, CD009354.

8. Begley, D.J., Pontikis, C.C., and Scarpa, M. (2008). Lysosomal storage diseases and the blood-brain barrier. Curr. Pharm. Des. *14*, 1566–1580.

9. Rohrbach, M., and Clarke, J.T.R. (2007). Treatment of lysosomal storage disorders: progress with enzyme replacement therapy. Drugs *67*, 2697–2716.

10. Valayannopoulos, V., and Wijburg, F.A. (2011). Therapy for the mucopolysaccharidoses. Rheumatology *50*, v49–v59.

11. Hampe, C.S., Wesley, J., Lund, T.C., Orchard, P.J., Polgreen, L.E., Eisengart, J.B., McLoon, L.K., Cureoglu, S., Schachern, P., and McIvor, R.S. (2021). Mucopolysaccharidosis Type I: Current Treatments, Limitations, and Prospects for Improvement. Biomolecules *11*, 189.

12. Penon-Portmann, M., Blair, D.R., and Harmatz, P. (2023). Current and new therapies for mucopolysaccharidoses. Pediatr. Neonatol. *64*, S10–S17. https://doi.org/10.1016/j.pedneo.2022.10.001.

13. Rodgers, N.J., Kaizer, A.M., Miller, W.P., Rudser, K.D., Orchard, P.J., and Braunlin, E.A. (2017). Mortality after hematopoietic stem cell transplantation for severe mucopolysaccharidosis type I: the 30-year University of Minnesota experience. J. Inherit. Metab. Dis. *40*, 271–280.

14. Aldenhoven, M., Jones, S.A., Bonney, D., Borrill, R.E., Coussons, M., Mercer, J., Bierings, M.B., Versluys, B., van Hasselt, P.M., Wijburg, F.A., et al. (2015). Hematopoietic cell transplantation for mucopolysaccharidosis patients is safe and effective: results after implementation of international guidelines. Biol. Blood Marrow Transplant. *21*, 1106–1109. https://doi.org/10.1016/j.bbmt.2015.02.011.

15. Braunlin, E., Miettunen, K., Lund, T., Luquette, M., and Orchard, P. (2019). Hematopoietic cell transplantation for severe MPS I in the first six months of life: The heart of the matter. Mol. Genet. Metab. *126*, 117–120.

16. Ziegler, R., and Shapiro, E. (2007). Metabolic and Neurodegenerative Diseases across the Life Span. In Principles and Practice of Lifespan Developmental Neuropsychology, J. Donders and S. Hunter, eds. (Cambridge University Press), pp. 427–448.

17. Gentner, B., Tucci, F., Galimberti, S., Fumagalli, F., De Pellegrin, M., Silvani, P., Camesasca, C., Pontesilli, S., Darin, S., Ciotti, F., et al. (2021). MPSI Study Group. Hematopoietic Stem- and Progenitor-Cell Gene Therapy for Hurler Syndrome. N. Engl. J. Med. *385*, 1929–1940.

18. Fumagalli, F., Calbi, V., Natali Sora, M.G., Sessa, M., Baldoli, C., Rancoita, P.M.V., Ciotti, F., Sarzana, M., Fraschini, M., Zambon, A.A., et al. (2022). Lentiviral haematopoietic stem-cell gene therapy for early-onset metachromatic leukodystrophy: long-term results from a non-randomised, open-label, phase 1/2 trial and expanded access. Lancet *399*, 372–383.

19. Wolf, D.A., Lenander, A.W., Nan, Z., Belur, L.R., Whitley, C.B., Gupta, P., Low, W.C., and McIvor, R.S. (2011). Direct gene transfer to the CNS prevents emergence of neurologic disease in a murine model of mucopolysaccharidosis type I. Neurobiol. Dis. *43*, 123–133.

20. Belur, L.R., Romero, M., Lee, J., Podetz-Pedersen, K.M., Nan, Z., Riedl, M.S., Vulchanova, L., Kitto, K.F., Fairbanks, C.A., Kozarsky, K.F., et al. (2021). Comparative Effectiveness of Intracerebroventricular, Intrathecal, and Intranasal Routes of AAV9 Vector Administration for Genetic Therapy of Neurologic Disease in Murine Mucopolysaccharidosis Type I. Front. Mol. Neurosci. *14*, 618360–618372.

21. Hurt, S.C., Dickson, P.I., and Curiel, D.T. (2021). Mucopolysaccharidoses type I gene therapy. J. Inherit. Metab. Dis. *44*, 1088–1098.

22. Belur, L.R., Podetz-Pedersen, K.M., Tran, T.A., Mesick, J.A., Singh, N.M., Riedl, M., Vulchanova, L., Kozarsky, K.F., and McIvor, R.S. (2020). Intravenous delivery for treatment of mucopolysaccharidosis type I: A comparison of AAV serotypes 9 and rh10. Mol. Genet. Metab. Rep. *24*, 100604–100612.

23. Belur, L.R., Temme, A., Podetz-Pedersen, K.M., Riedl, M., Vulchanova, L., Robinson, N., Hanson, L.R., Kozarsky, K.F., Orchard, P.J., Frey, W.H., 2nd, et al. (2017). Intranasal Adeno-Associated Virus Mediated Gene Delivery and Expression of Human Iduronidase in the Central Nervous System: A Noninvasive and Effective Approach for Prevention of Neurologic Disease in Mucopolysaccharidosis Type I. Hum. Gene Ther. *28*, 576–587.

24. Hinderer, C., Bell, P., Louboutin, J.P., Zhu, Y., Yu, H., Lin, G., Choa, R., Gurda, B.L., Bagel, J., O'Donnell, P., et al. (2015). Neonatal Systemic AAV Induces Tolerance to CNS Gene Therapy in MPS I Dogs and Nonhuman Primates. Mol. Ther. *23*, 1298–1307.

25. Hartung, S.D., Frandsen, J.L., Pan, D., Koniar, B.L., Graupman, P., Gunther, R., Low, W.C., Whitley, C.B., and McIvor, R.S. (2004). Correction of metabolic, craniofacial, and neurologic abnormalities in MPS I mice treated at birth with adeno-associated virus vector transducing the human alpha-L-iduronidase gene. Mol. Ther. *9*, 866–875.

26. Hinderer, C., Bell, P., Gurda, B.L., Wang, Q., Louboutin, J.P., Zhu, Y., Bagel, J., O'Donnell, P., Sikora, T., Ruane, T., et al. (2014). Liver-directed gene therapy corrects cardiovascular lesions in feline mucopolysaccharidosis type I. Proc. Natl. Acad. Sci. USA *111*, 14894–14899.

27. Hinderer, C., Bell, P., Gurda, B.L., Wang, Q., Louboutin, J.P., Zhu, Y., Bagel, J., O'Donnell, P., Sikora, T., Ruane, T., et al. (2014). Intrathecal gene therapy corrects CNS pathology in a feline model of mucopolysaccharidosis I. Mol. Ther. *22*, 2018–2027.

28. Hordeaux, J., Hinderer, C., Buza, E.L., Louboutin, J.P., Jahan, T., Bell, P., Chichester, J.A., Tarantal, A.F., and Wilson, J.M. (2019). Safe and Sustained Expression of Human Iduronidase After Intrathecal Administration of Adeno-Associated Virus Serotype 9 in Infant Rhesus Monkeys. Hum. Gene Ther. *30*, 957–966.

29. Hordeaux, J., Hinderer, C., Goode, T., Katz, N., Buza, E.L., Bell, P., Calcedo, R., Richman, L.K., and Wilson, J.M. (2018). Toxicology Study of Intra-Cisterna Magna Adeno-Associated Virus 9 Expressing Human Alpha-L-Iduronidase in Rhesus Macaques. Mol. Ther. Methods Clin. Dev. *10*, 79–88.

30. Watson, G., Bastacky, J., Belichenko, P., Buddhikot, M., Jungles, S., Vellard, M., Mobley, W.C., and Kakkis, E. (2006). Intrathecal administration of AAV vectors for the treatment of lysosomal storage in the brains of MPS I mice. Gene Ther. *13*, 917–925.

31. Ou, L., DeKelver, R.C., Rohde, M., Tom, S., Radeke, R., St Martin, S.J., Santiago, Y., Sproul, S., Przybilla, M.J., Koniar, B.L., et al. (2019). ZFN-Mediated In Vivo Genome Editing Corrects Murine Hurler Syndrome. Mol. Ther. *27*, 178–187.

32. Podetz-Pedersen, K.M., Laoharawee, K., Singh, S., Nguyen, T.T., Smith, M.C., Temme, A., Kozarsky, K., McIvor, R.S., and Belur, L.R. (2023). Neurologic Recovery in MPS I and MPS II Mice by AAV9-Mediated Gene Transfer to the CNS After the Development of Cognitive Dysfunction. Hum. Gene Ther. *34*, 8–18.

33. Hocquemiller, M., Hemsley, K.M., Douglass, M.L., Tamang, S.J., Neumann, D., King, B.M., Beard, H., Trim, P.J., Winner, L.K., Lau, A.A., et al. (2020). AAVrh10 Vector Corrects Disease Pathology in MPS IIIA Mice and Achieves Widespread Distribution of SGSH in Large Animal Brains. Mol. Ther. Methods Clin. Dev. *17*, 174–187.

34. Marcó, S., Haurigot, V., and Bosch, F. (2019). *In Vivo* Gene Therapy for Mucopolysaccharidosis Type III (Sanfilippo Syndrome): A New Treatment Horizon. Hum. Gene Ther. *30*, 1211–1221.

35. Ciron, C., Cressant, A., Roux, F., Raoul, S., Cherel, Y., Hantraye, P., Déglon, N., Schwartz, B., Barkats, M., Heard, J.M., et al. (2009). Human α- iduronidase gene transfer mediated by adeno-associated virus types 1, 2, and 5 in the brain of nonhuman primates: vector diffusion and bio-distribution. Hum. Gene Ther. *20*, 350–360.

36. Desmaris, N., Verot, L., Puech, J.P., Caillaud, C., Vanier, M.T., and Heard, J.M. (2004). Prevention of neuropathology in the mouse model of Hurler syndrome. Ann. Neurol. *56*, 68–76.

37. Ellinwood, N.M., Ausseil, J., Desmaris, N., Bigou, S., Liu, S., Jens, J.K., Snella, E.M., Mohammed, E.E.A., Thomson, C.B., Raoul, S., et al. (2011). Safe, efficient, and reproducible gene therapy of the brain in the dog models of Sanfilippo and Hurler syndromes. Mol. Ther. *19*, 251–259.

38. Laoharawee, K., Podetz-Pedersen, K.M., Nguyen, T.T., Evenstar, L.B., Kitto, K.F., Nan, Z., Fairbanks, C.A., Low, W.C., Kozarsky, K.F., and McIvor, R.S. (2017). Prevention of Neurocognitive Deficiency in Mucopolysaccharidosis Type II Mice by Central Nervous System-Directed, AAV9-Mediated Iduronate Sulfatase Gene Transfer. Hum. Gene Ther. *28*, 626–638.

39. Bailey, R.M., Rozenberg, A., and Gray, S.J. (2020). Comparison of high-dose intracisterna magna and lumbar puncture intrathecal delivery of AAV9 in mice to treat neuropathies. Brain Res. *1739*, 146832. https://doi.org/10.1016/j.brainres.2020.146832.

40. Taghian, T., Marosfoi, M.G., Puri, A.S., Cataltepe, O.I., King, R.M., Diffie, E.B., Maguire, A.S., Martin, D.R., Fernau, D., Batista, A.R., et al. (2020). A Safe and Reliable Technique for CNS Delivery of AAV Vectors in the Cisterna Magna. Mol. Ther. *28*, 411–421.

41. Hordeaux, J., Jeffrey, B.A., Jian, J., Choudhury, G.R., Michalson, K., Mitchell, T.W., Buza, E.L., Chichester, J., Dyer, C., Bagel, J., et al. (2022). Efficacy and Safety of a Krabbe Disease Gene Therapy. Hum. Gene Ther. *33*, 499–517.

42. Fu, H., Kang, L., Jennings, J.S., Moy, S.S., Perez, A., Dirosario, J., McCarty, D.M., and Muenzer, J. (2007). Significantly increased lifespan and improved behavioral performances by rAAV gene delivery in adult mucopolysaccharidosis IIIB mice. Gene Ther. *14*, 1065–1077.

43. Ferla, R., Alliegro, M., Marteau, J.B., Dell'Anno, M., Nusco, E., Pouillot, S., Galimberti, S., Valsecchi, M.G., Zuliani, V., and Auricchio, A. (2017). Non-clinical Safety and Efficacy of an AAV2/8 Vector Administered Intravenously for Treatment of Mucopolysaccharidosis Type VI. Mol. Ther. Methods Clin. Dev. *6*, 143–158.

44. Laoharawee, K., Podetz-Pedersen, K.M., Nguyen, T.T., Singh, S.M., Smith, M.C., Belur, L.R., Low, W.C., Kozarsky, K.F., and McIvor, R.S. (2023). Non-invasive intravenous administration of AAV9 transducing iduronate sulfatase leads to global metabolic correction and prevention of neurologic deficits in a mouse model of Hunter syndrome. Mol. Genet. Metab. Rep. *34*, 100956.

45. Bertolin, J., Sánchez, V., Ribera, A., Jaén, M.L., Garcia, M., Pujol, A., Sánchez, X., Muñoz, S., Marcó, S., Pérez, J., et al. (2021). Treatment of skeletal and non-skeletal alterations of Mucopolysaccharidosis type IVA by AAV-mediated gene therapy. Nat. Commun. *12*, 5343.

46. Meyer, K., Ferraiuolo, L., Schmelzer, L., Braun, L., McGovern, V., Likhite, S., Michels, O., Govoni, A., Fitzgerald, J., Morales, P., et al. (2015). Improving single injection CSF delivery of AAV9-mediated gene therapy for SMA: a dose-response study in mice and nonhuman primates. Mol. Ther. *23*, 477–487.

47. Samaranch, L., Salegio, E.A., San Sebastian, W., Kells, A.P., Foust, K.D., Bringas, J.R., Lamarre, C., Forsayeth, J., Kaspar, B.K., and Bankiewicz, K.S. (2012). Adeno-associated virus serotype 9 transduction in the central nervous system of nonhuman primates. Hum. Gene Ther. *23*, 382–389.

48. Braunlin, E., Mackey-Bojack, S., Panoskaltsis-Mortari, A., Berry, J.M., McElmurry, R.T., Riddle, M., Sun, L.Y., Clarke, L.A., Tolar, J., and Blazar, B.R. (2006). Cardiac functional and histopathologic findings in humans and mice with mucopolysaccharidosis type I: implications for assessment of therapeutic interventions in hurler syndrome. Pediatr. Res. *59*, 27–32.

49. Tolar, J., Braunlin, E., Riddle, M., Peacock, B., McElmurry, R.T., Orchard, P.J., and Blazar, B.R. (2009). Gender-related dimorphism in aortic insufficiency in murine mucopolysaccharidosis type I. J. Heart Valve Dis. *18*, 524–529.

50. Braunlin, E., Abrahante, J.E., McElmurry, R., Evans, M., Smith, M., Seelig, D., O'Sullivan, M.G., Tolar, J., Whitley, C.B., and McIvor, R.S. (2022). Contribution of the innate and adaptive immune systems to aortic dilation in murine mucopolysaccharidosis type I. Mol. Genet. Metab. *135*, 193–205.

51. Davidoff, A.M., Ng, C.Y.C., Zhou, J., Spence, Y., and Nathwani, A.C. (2003). Sex significantly influences transduction of murine liver by recombinant adeno-associated viral vectors through an androgen-dependent pathway. Blood *102*, 480–488.

52. Weismann, C.M., Ferreira, J., Keeler, A.M., Su, Q., Qui, L., Shaffer, S.A., Xu, Z., Gao, G., and Sena-Esteves, M. (2015). Systemic AAV9 gene transfer in adult GM1 gangliosidosis mice reduces lysosomal storage in CNS and extends lifespan. Hum. Mol. Genet. *24*, 4353–4364.

53. Maguire, C.A., Crommentuijn, M.H., Mu, D., Hudry, E., Serrano-Pozo, A., Hyman, B.T., and Tannous, B.A. (2013). Mouse gender influences brain transduction by intravascularly administered AAV9. Mol. Ther. *21*, 1470–1471.

54. Piechnik, M., Amendum, P.C., Sawamoto, K., Stapleton, M., Khan, S., Fnu, N., Álvarez, V., Pachon, A.M.H., Danos, O., Bruder, J.T., et al. (2022). Sex Difference Leads to Differential Gene Expression Patterns and Therapeutic Efficacy in Mucopolysaccharidosis IVA Murine Model Receiving AAV8 Gene Therapy. Int. J. Mol. Sci. *23*, 12693.

55. Ou, L., Herzog, T., Koniar, B.L., Gunther, R., and Whitley, C.B. (2014). High-dose enzyme replacement therapy in murine Hurler syndrome. Mol. Genet. Metab. *111*, 116–122.

56. Laoharawee, K., DeKelver, R.C., Podetz-Pedersen, K.M., Rohde, M., Sproul, S., Nguyen, H.O., Nguyen, T., St Martin, S.J., Ou, L., Tom, S., et al. (2018). Dose-Dependent Prevention of Metabolic and Neurologic Disease in Murine MPS II by ZFN-Mediated In Vivo Genome Editing. Mol. Ther. *26*, 1127–1136.

57. Au, H.K.E., Isalan, M., and Mielcarek, M. (2021). Gene Therapy Advances: A Meta-Analysis of AAV Usage in Clinical Settings. Front. Med. *8*, 809118.

58. Servais, L., Day, J.W., De Vivo, D.C., Kirschner, J., Mercuri, E., Muntoni, F., Proud, C.M., Shieh, P.B., Tizzano, E.F., Quijano-Roy, S., et al. (2024). Real-World Outcomes in Patients with Spinal Muscular Atrophy Treated with Onasemnogene Abeparvovec Monotherapy: Findings from the RESTORE Registry. J. Neuromuscul. Dis. *11*, 425.

59. Mendell, J.R., Al-Zaidy, S., Shell, R., Arnold, W.D., Rodino-Klapac, L.R., Prior, T.W., Lowes, L., Alfano, L., Berry, K., Church, K., et al. (2017). Single-Dose Gene-Replacement Therapy for Spinal Muscular Atrophy. N. Engl. J. Med. *377*, 1713–1722.

60. Wilson, J.M., and Flotte, T.R. (2020). Moving Forward After Two Deaths in a Gene Therapy Trial of Myotubular Myopathy. Hum. Gene Ther. *31*, 695–696.

61. Manno, C.S., Pierce, G.F., Arruda, V.R., Glader, B., Ragni, M., Rasko, J.J., Ozelo, M.C., Hoots, K., Blatt, P., Konkle, B., et al. (2006). Successful transduction of liver in hemophilia by AAV-Factor IX and limitations imposed by the host immune response. Nat. Med. *12*, 342–347. Erratum in: Nat Med. 2006 May;12(5):592. Rasko, John [corrected to Rasko, John JE]; Rustagi, Pradip K [added].

62. George, L.A., Sullivan, S.K., Giermasz, A., Rasko, J.E.J., Samelson-Jones, B.J., Ducore, J., Cuker, A., Sullivan, L.M., Majumdar, S., Teitel, J., et al. (2017). Hemophilia B Gene Therapy with a High-Specific-Activity Factor IX Variant. N. Engl. J. Med. *377*, 2215–2227.

63. Smith, M.C., Belur, L.R., Karlen, A.D., Erlanson, O., Furcich, J., Lund, T.C., Seelig, D., Kitto, K.F., Fairbanks, C.A., Kim, K.H., et al. (2024). Comparative dose effectiveness of intravenous and intrathecal AAV9.CB7.hIDS, RGX-121, in mucopolysaccharidosis type II mice. Mol. Ther. Methods Clin. Dev. *32*, 101201.

64. Garcia-Rivera, M.F., Colvin-Wanshura, L.E., Nelson, M.S., Nan, Z., Khan, S.A., Rogers, T.B., Maitra, I., Low, W.C., and Gupta, P. (2007). Characterization of an immunodeficient mouse model of mucopolysaccharidosis type I suitable for preclinical testing of human stem cell and gene therapy. Brain Res. Bull. *74*, 429–438.

65. Barnes, C.A. (1979). Memory deficits associated with senescence: a neurophysiological and behavioral study in the rat. J. Comp. Physiol. Psychol. *93*, 74–104.

OMTM, Volume *32*

Supplemental information

Intrathecal or intravenous AAV9-IDUA/RGX-111

at minimal effective dose prevents cardiac, skeletal

and neurologic manifestations of murine MPS I

Lalitha R. Belur, Avery K. Huber, Hillary Mantone, Mason Robertson, Miles C. Smith, Andrea D. Karlen, Kelley F. Kitto, Li Ou, Chester B. Whitley, Elizabeth Braunlin, Justin Furcich, Troy C. Lund, Davis Seelig, Carolyn A. Fairbanks, Nicholas Buss, Kwi Hye Kim, and R. Scott McIvor

SUPPLEMENTAL FIGURES.

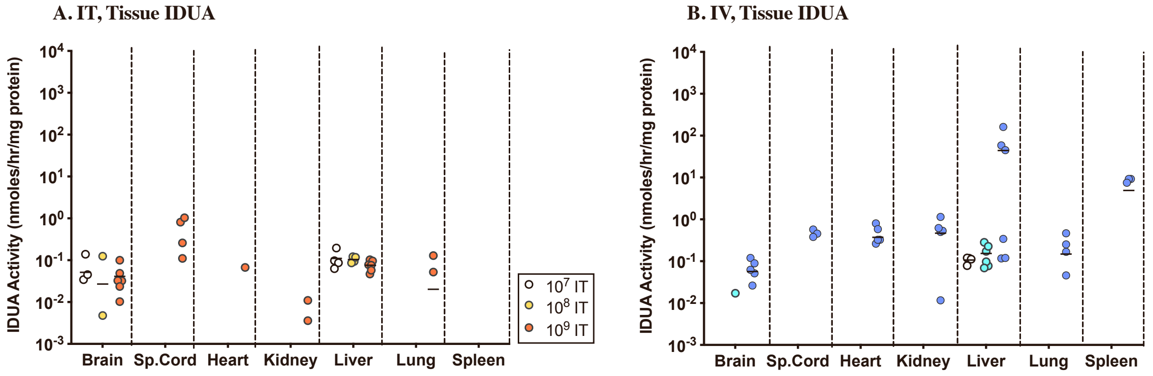

Figure S1. Tissue IDUA activities in MPS mice administered 10^7-10^9 vg RGX-111. ***(Most related to Figure 2; Tissue IDUA Activities)*** **(A)** Intrathecal (IT) administration **(B)** Intravenous (IV) administration. Each data point indicates a value from a single animal. Tissues are indicated across the bottom of the graph. Route of administration or control group are indicated in the key. N = 6 (3 males and 3 females).

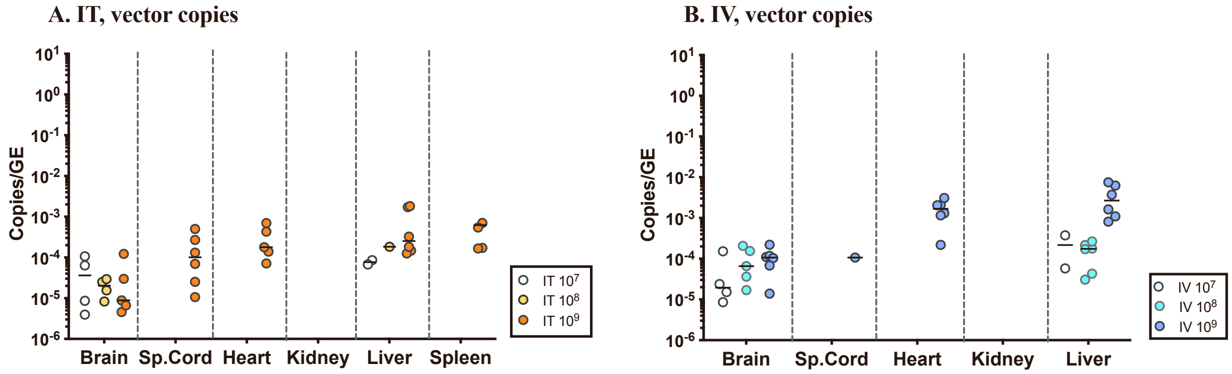

Figure S2. Biodistribution by qPCR for IDUA vector sequences ***(Most related to Figure 2; Vector distribution by qPCR).*** Tissue DNA extracts from animals treated with 10^7-10^9 vg RGX-111 either IT **(A)** or IV **(B)** were assayed for the presence of IDUA sequences by quantitative PCR. Dose is indicated in the key. Each symbol represents 1 animal, with the mean indicated by a horizontal bar. The lower limit of detection was determined by analysis of genomic DNA samples collected from heterozygote controls (<0.001 vector copies/genome equivalent).

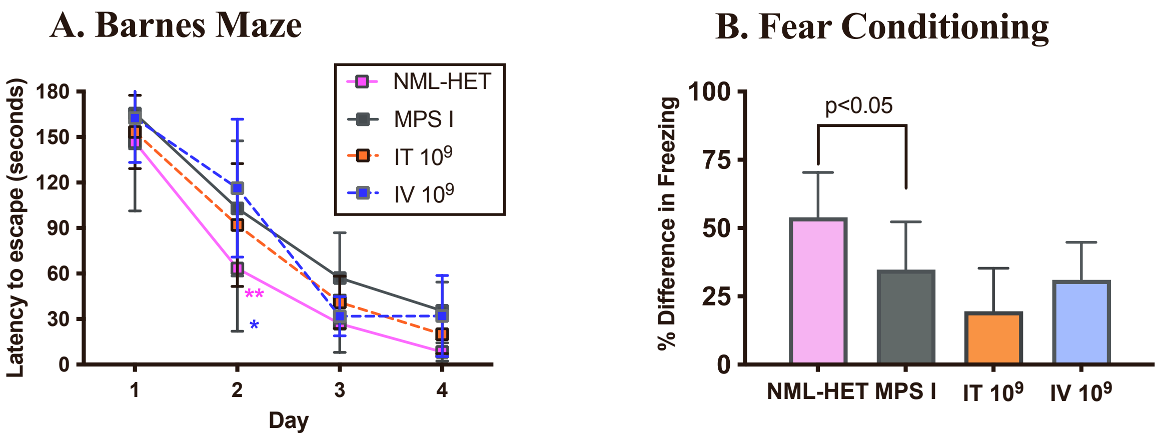

Figure S3. Effect of RGX-111 treatment on neurocognition *(Most related to Figure 3; Barnes Maze and Fear Conditioning)*. Animals treated with 10^9 vg RGX-111 either IT or IV were assayed for neurobehavior. **(A) Barnes maze.** The Barnes maze was used to assess the effect of vector administration on spatial learning and memory. Testing was carried out in four trials per day over 4 days for all groups (N=6) **P<.01 *P<.05. Mean latency to escape for the 4 trials is a shown for each day. **(B) Fear conditioning.** All groups were evaluated for fear conditioned response. Mean freezing time as a cued response is shown (N=6).

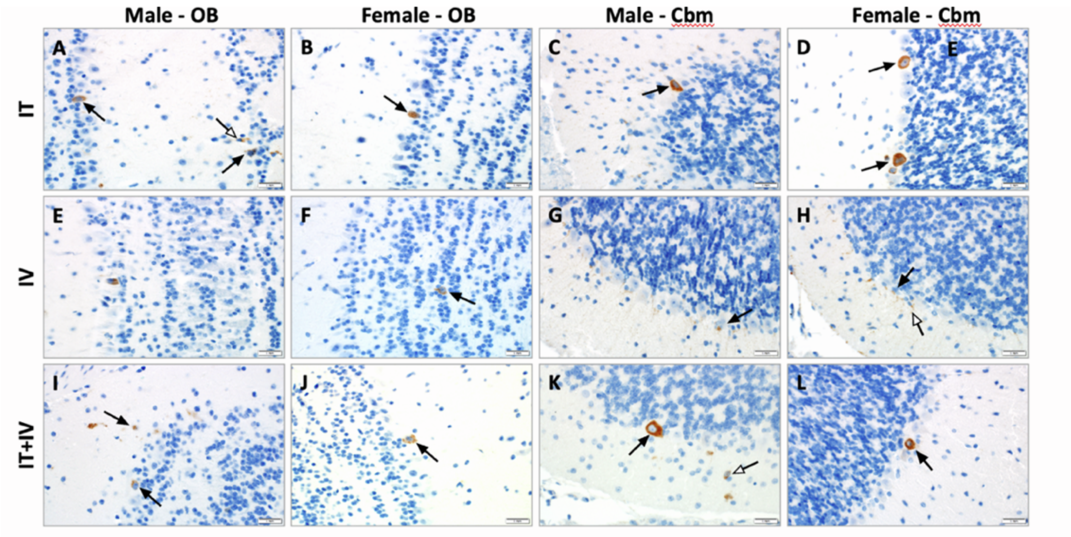

Figure S4. Similar density of IDUA-positive cells in the brains of male and female mice treated with 10^{10} vg RGX-111 (*Most related to Figure 7; IDUA-positive cells in livers of male and female mice*). Irrespective of sex, all treated mice demonstrate low numbers of IDUA-positive neurons (black arrows) and presumed glial cells (white arrows). Representative images from the olfactory bulb (OB) and the cerebellum (Cbm). Scale bars: A-L = 20 µm
